# Redundant and Distinct Roles of Two 14-3-3 Proteins in *Fusarium sacchari*, Pathogen of Sugarcane Pokkah Boeng Disease

**DOI:** 10.3390/jof10040257

**Published:** 2024-03-28

**Authors:** Yuejia Chen, Ziting Yao, Lixian Zhao, Mei Yu, Baoshan Chen, Chengwu Zou

**Affiliations:** 1 State Key Laboratory for Conservation and Utilization of Subtropical Agro-Bioresources, Ministry & Province Co-Sponsored Center of Collaborative Innovation for Sugarcane Industry, College of Life Science and Technology, Guangxi University, Nanning 530004, China; 18172258103@163.com (Y.C.); 15677109451@163.com (M.Y.); 2 Plant Protection Research Institute, Guangxi Academy of Agriculture Science, Nanning 530007, China; yaoziting@gxaas.net; 3 Guangxi Key Laboratory of Sugarcane Biology, College of Agriculture, Guangxi University, Nanning 530004, China; zhaolx513@163.com

**Keywords:** *Fusarium sacchari*, Pokkah boeng disease, 14-3-3 proteins, *FsBmh*, hyphal growth, sporulation, virulence, transcriptome, interaction

## Abstract

*Fusarium sacchari*, a key pathogen of sugarcane, is responsible for the Pokkah boeng disease (PBD) in China. The 14-3-3 proteins have been implicated in critical developmental processes, including dimorphic transition, signal transduction, and carbon metabolism in various phytopathogenic fungi. However, their roles are poorly understood in *F. sacchari.* This study focused on the characterization of two 14-3-3 protein-encoding genes, *FsBmh1* and *FsBmh2*, within *F. sacchari*. Both genes were found to be expressed during the vegetative growth stage, yet *FsBmh1* was repressed at the sporulation stage in vitro. To elucidate the functions of these genes, the deletion mutants ΔFsBmh1 and ΔFsBmh2 were generated. The ΔFsBmh2 exhibited more pronounced phenotypic defects, such as impaired hyphal branching, septation, conidiation, spore germination, and colony growth, compared to the ΔFsBmh1. Notably, both knockout mutants showed a reduction in virulence, with transcriptome analysis revealing changes associated with the observed phenotypes. To further investigate the functional interplay between *FsBmh1* and *FsBmh2*, we constructed and analyzed mutants with combined deletion and silencing (ΔFsBmh/siFsBmh) as well as overexpression (O-FsBmh). The combinations of ΔFsBmh1/siFsBmh2 or ΔFsBmh2/siFsBmh1 displayed more severe phenotypes than those with single allele deletions, suggesting a functional redundancy between the two 14-3-3 proteins. Yeast two-hybrid (Y2H) assays identified 20 proteins with pivotal roles in primary metabolism or diverse biological functions, 12 of which interacted with both FsBmh1 and FsBmh2. Three proteins were specifically associated with FsBmh1, while five interacted exclusively with FsBmh2. In summary, this research provides novel insights into the roles of *FsBmh1* and *FsBmh2* in *F. sacchari* and highlights potential targets for PBD management through the modulation of *FsBmh* functions.

## 1. Introduction

Sugarcane Pokkah boeng disease (PBD) is a pervasive airborne fungal disease caused by the *Fusarium* species complex (FSC) [1,2]. The disease is characterized by the dissemination of fungal spores via air and rain, which infect the sugarcane shoot tips, leading to symptoms such as leaf yellowing, crinkling, and twisting, with severe cases resulting in tip rot [3]. In China, *F. sacchari* emerges as the predominant pathogen responsible for PBD [4]. Despite extensive research encompassing pathogen identification, germplasm resistance, environmental factor influences, and pathogen effectors [5,6,7], the pathogenic mechanisms of *F. sacchari* are still far from clear.

The 14-3-3 protein family, with a low molecular weight of approximately 30 kDa, is ubiquitously present in eukaryotic organisms [8,9]. These proteins exert their influence by binding to target proteins, thereby modulating their activity, localization, and presentation, to modulate a wide variety of cellular processes [10,11,12,13]. Within the cell, 14-3-3 proteins typically exist as homodimers or heterodimers [14]. Isoforms are prevalent within this family, with examples including 15 isoforms in the plant Arabidopsis and seven in mammals [15], while fungi typically exhibit a lesser number, such as one or two [16,17]. Despite high conservation among isoforms, distinct isoforms may exhibit varying affinities for their preferred targets, thus executing different functions [18].

In *Saccharomyces cerevisiae*, the 14-3-3-encoding genes *Bmh1* and *Bmh2* are pivotal in regulating vegetative growth, and the knockout of both genes results in a lethal phenotype [19]. Similarly, in the human pathogen *Candida albicans*, *Bmh1* is crucial for growth, morphogenesis, and pathogenesis [20]. In the plant pathogen *Ustilago maydis*, *Pdc1*, which encodes a homolog of Bmh1 from yeast, plays a role in the regulation of mycelial growth and virulence [21]. In *Fusarium graminearum*, two 14-3-3 proteins encoding genes have partially overlapping roles in mediating nitrogen catabolite repression, yet only *FgBmh2* is implicated in the regulation of reproduction and virulence [22]. However, the functional role of 14-3-3 proteins in *F. sacchari* has remained unexplored. This study delves into the functions of the 14-3-3 protein-encoding genes *FsBmh1* and *FsBmh2* in *F. sacchari* using a comprehensive approach. The findings reveal that both genes are essential for virulence and exhibit redundancy in many functions, with *FsBmh2* playing a predominant role in the regulation of mycelial growth and conidiation in *F. sacchari*.

## 2. Materials and Methods

### 2.1. Fungal Strains and Culturing Conditions

The wild-type strain CNO-1 of *F. sacchari* and mutant strains were stored in 25% (*v*/*v*) glycerin at −80 °C. The phenotypic characteristics, including the colony diameter and pigmentation, were monitored on potato dextrose agar (PDA) medium at 28 °C for 7 days [23].

### 2.2. Generation of Mutant Strains

To knockout *FsBmh1* and *FsBmh2*, deletion fragments were amplified using fusion PCR with the primer pairs FsBmh1-1F/R, FsBmh2-1F/R, FsBmh1-2F/R, FsBmh2-2F/R, Hph-F/R, and G418-F/R (Table S1). The hygromycin resistance gene (*hph*) and the geneticin resistance gene (*NeoR*) were employed as selection markers [24]. The amplified fragments were transformed into protoplasts of the wild-type strain CNO-1 using a polyethylene glycol (PEG)-mediated method, resulting in the creation of ΔFsBmh1 and ΔFsBmh2 mutants, respectively [25]. For the complementation mutant strains, full-length *FsBmh1* and *FsBmh2* fragments, including their promoter sequences, were amplified using the primer pairs C-FsBmh1-F/R and C-FsBmh2-F/R (Table S1). These fragments were cloned into the pCPXG418 or pCPXHY2 vector using the pEASY-Basic Seamless Cloning and Assembly Kit (Transgen Biotech, Beijing, China) and then transformed into the protoplasts of ΔFsBmh1 or ΔFsBmh2 mutants.

RNA interference was utilized to silence *FsBmh1* in the ΔFsBmh2 background or vice versa [26]. The interference fragments for *FsBmh1* and *FsBmh2*, 520 bp and 519 bp, respectively, were amplified from the genomic DNA of the wild-type strain and cloned into the pCPXG418 or pCPXHY2 vector using the pEASY-Basic Seamless Cloning and Assembly Kit. The overexpression strains were developed by introducing additional copies of *FsBmh1* or *FsBmh2* into the CNO-1 protoplasts, which were then selected on media supplemented with the appropriate antibiotics.

### 2.3. RNA Extraction and Quantitative Real-Time RT-PCR

The total RNA from each strain was extracted using the RNA extraction kit (TaKaRa, Beijing, China) according to the manufacturer’s protocol. First-strand cDNA synthesis was performed using the FastQuant RT Kit (TaKaRa, Beijing, China). Quantitative real-time PCR was conducted using the SuperReal PreMix Plus (SYBR Green) (TaKaRa, Beijing, China) with the target gene primer pairs (Table S2) and 18S rRNA as an internal control. The relative expression levels were calculated using the 2^−ΔΔCT^ method [27]. The data are presented as the mean ± standard deviation from three independent biological replicates, each with three technical repeats.

### 2.4. Induction of Macrospores

Carnation leaf agar is often used to induce macrospore production in *Fusarium* spp. CLA medium was prepared by aseptically placing sterile carnation leaf pieces, 3–5 mm^2^, into a dish and adding sterile water agar (20 g agar in 1 L sterile water) [28]. Macroconidia were induced by inoculating mycelial plugs on carnation leaf agar for 7 days.

### 2.5. Microscopy

Microconidia from each strain were harvested from PDA plates or macroconidia from carnation leaf agar plates using 10 mL of sterile water. Images were captured using a differential-interference contrast microscope.

The hyphae and spores were stained with calcofluor white (CFW) for septa observation [29]. The sugarcane tissue samples were stained with 0.05% of aniline blue, which specifically binds the fungal cell to allow it to be visualized in a blue color to trace the development of mycelium [30]. The samples were viewed under an Olympus DP70 microscope (Olympus, Tokyo, Japan).

### 2.6. Pathogenicity Assays

Susceptible sugarcane seedlings (Zhongzhe 9, ZZ9) were grown in a greenhouse until the 5-leaf stage. *F. sacchari* conidia from 7-days-old PDA cultures were adjusted to 1 × 10^4^ conidia/mL. For inoculation, a volume of 300 µL of spore suspension was injected into the sugarcane stem around the tip meristem using a sterile needle. The disease severity was evaluated 21 days post-inoculation (dpi), and the disease severity index (DSI) was calculated using a symptom severity scale (Table S3). The DSI was determined using the formula DSI = 100 × (Σ score/5*N*), where *N* is the number of observed seedlings (*N* = 100). Statistical analysis was performed using one-way ANOVA with SPSS 23.0 software, and each assay was replicated three times.

### 2.7. Transcriptomic Analysis

The strains of interest were cultured in potato dextrose broth (PDB) medium at 28 °C and 200 rpm for 3 days. The mycelium was harvested and washed, and the total RNA was extracted using TRIzol^®^Reagent (Invitrogen, San Diego, CA, USA) with genomic DNA removal via DNase I (TaKaRa, Beijing, China) treatment. Transcriptome libraries were constructed using the TruSeq^TM^ RNA sample preparation Kit (Illumina, San Diego, CA, USA) and sequenced on the Illumina NovaSeq 6000 platform. The raw reads were filtered and mapped to the *F. sacchari* CNO-1 genome (unpublished data) using TopHat2 v2.1.1. Differentially expressed genes (DEGs) were identified based on a minimum two-fold change in expression (log2 ratio ≤ −1 or ≥1) at a false discovery rate of *p* = 0.05 or less [31]. DEGs were annotated using the NCBI protein databases for *Fusarium* spp., and KEGG pathway enrichment analysis was performed for all the DEGs with a corrected *p*-adjust < 0.05 [32]. Phenotype-associated gene transcripts were verified using cDNA from transcriptome samples and the fungal 18S RNA gene as an internal standard. Data were obtained from three biological replicates.

### 2.8. Yeast Two-Hybrid Assays

For Y2H analysis, the coding sequences of the genes under investigation were amplified from CNO-1 cDNA using the primer pairs listed in Table S12. These fragments were cloned into the yeast GAL4-binding domain vector pGBKT7 and GAL4 activation vector pGADT7 (TaKaRa, Beijing, China). The Y2H constructs were co-transformed into *S. cerevisiae* Y2H gold (Coolaber, Beijing, China) following the lithium acetate/single-stranded DNA/polyethylene glycol transformation protocol [33]. Positive and negative controls were included, and the transformants were cultured on synthetic medium lacking Leu and Trp, followed by transfer to medium lacking His, Ade, Leu, and Trp. The Y2H assay results were confirmed using three independent experiments.

### 2.9. Statistical Analysis

Statistical analysis was conducted using one-way ANOVA with the SPSS statistical package version 23.0 (IBM, Amonk, NY, USA). The Student–Newman–Keuls test was employed for inter-treatment comparisons at a significance level of *p* < 0.05.

## 3. Results

### 3.1. Conservation of 14-3-3 Proteins in Fusarium Species

Two distinct genes (*FsBmh1* and *FsBmh2*) both encoding 14-3-3 proteins were identified in the CNO-1 genome by blast of the *F. sacchari* genome database (our unpublished data) using the *S. cerevisiae* Bmh1 and Bmh2 as queries. The cDNAs of *FsBmh1* (FVER_07211, NCBI accession No. MH999450.1) and *FsBmh2* (FVER_01000, NCBI accession No. MH999451.1) were 807 bp and 849 bp, encoding 268 and 276 amino acids, respectively. These proteins possess the characteristic functional domain of the 14-3-3 protein superfamily and exhibit 69.6% identity and 83.4% similarity at the amino acid level. Notably, the amino acid identity within the *Fusarium* genus (*F. graminearum*, *F. fujikuroi*, and *F. oxysporum*) ranged from 91.4% to 97%, whereas the identity between different fungal genera was significantly lower, such as *S. cerevisiae* (62.4%) and *C. albicans* (73.1%) (Figure S1A). Phylogenetic analysis revealed that Bmh1 and Bmh2 segregate into distinct clusters among fungal species, with the *F. sacchari*-derived Bmh1 and Bmh2 showing the closest phylogenetic relationship to their counterparts in *F. fujikuroi* and *F. oxysporum*, respectively (Figure S1B).

### 3.2. Expression Patterns of FsBmh1 and FsBmh2 during Infection and Vegetative Growth

**Figure 1:**
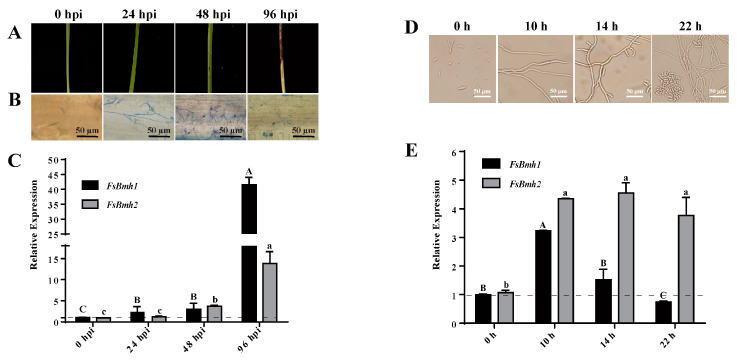
Expression patterns of *FsBmh1* and *FsBmh2* at different developmental stages in vivo and in vitro. (**A**) Symptom development. Sugarcane seedlings were inoculated with CNO-1. (**B**) Development of infection. Tissue sections were stained with aniline blue to trace fungal hyphae development. Samples were observed under a light microscope, scale bar = 50 µm. (**C**) Relative expression of *FsBmh1* and *FsBmh2* during infection. (**D**) Development of hyphae and asexual spores on liquid medium. The spore suspension of CNO-1 was inoculated in PDB medium, and representative pictures were photographed at 10 h, 14 h, and 22 h by microscope, scale bar = 50 µm. (**E**) Relative expression of *FsBmh1* and *FsBmh2* during saprophytic stages. The expression of *FsBmh1* and *FsBmh2* transcripts was measured by quantitative real-time RT-PCR (2^−ΔΔCT^method) with 18S rRNA as internal reference. The transcript levels of 0 h post-inoculation were set to a value of 1.0 and indicated by the dotted line. Different letters indicate significant differences at *p* < 0.05.

Sugarcane seedlings were inoculated with the spore suspension, and the invasion of the plant by hyphae was observed within 24 h post-inoculation (hpi). Hyphal branching, plant cell disintegration, and necrotic spots on the leaf surface were evident at 48 hpi, with severe tissue necrosis, hyphae proliferation within infected cells, and new spore formation observed at 96 hpi (Figure 1A,B). In concert with these developmental stages, the transcript level of *FsBmh1* increased by 2-3-fold at 24 and 48 hpi and sharply lifted by 41-fold at 96 hpi compared to 0 hpi; the *FsBmh2* transcript levels remained unchanged during the initial 24 hpi but increased by approximately 2-fold at 48 hpi and 15-fold at 96 hpi (Figure 1C). Although both *FsBmh1* and *FsBmh2* exhibited transcriptional responses during host colonization, *FsBmh1* responded earlier and at a significantly higher level at the later stage (96 hpi) in planta compared to *FsBmh2*.

To explore the correlation between the developmental timeline and transcriptional dynamics of *FsBmh* in a saprophytic context, *F. sacchari* spores were inoculated into potato dextrose broth (PDB) medium. Initial hyphal growth was observed at 10 h, branching at 14 h, and new spore formation at 22 h (Figure 1D). Transcript level analysis indicated that *FsBmh1* was up-regulated by 3.3-fold at 10 h, followed by a decline; in contrast, *FsBmh2* was up-regulated by 4.3-fold at 10 h and maintained a relatively stable level thereafter (Figure 1E). A comparative analysis of the transcription profiles (Figure 1C vs. Figure 1E) revealed distinct transcriptional dynamics for *FsBmh1* and *FsBmh2* during plant infection and vegetative growth in the medium.

### 3.3. The Expression of FsBmh1 and FsBmh2 Are Mutually Compensated in Cells

**Figure 2:**
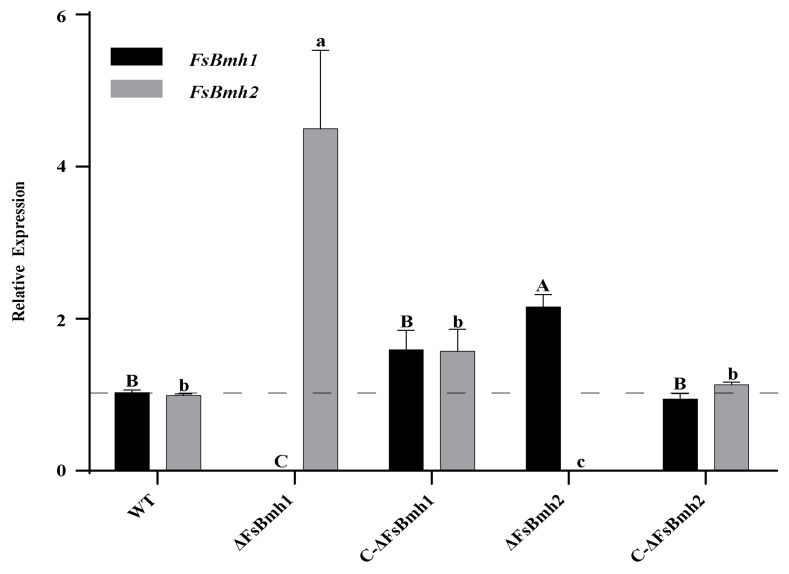
Validation of *FsBmh1* and *FsBmh2* expression using qRT-PCR in *FsBmh* deletion and complementation mutants. Relative expression of *FsBmh* genes in *FsBmh-*deletion and complementation mutants were determined by qRT-PCR. The expression of *FsBmh1* and *FsBmh2* transcripts was measured by quantitative real-time RT-PCR (2^−ΔΔCT^ method) with 18S rRNA as internal reference. The transcript levels of WT were set to a value of 1.0 and indicated by the dotted line. Different letters indicate significant differences at *p* < 0.05.

To elucidate the distinct functions of two 14-3-3 proteins, we generated the gene deletion mutants ΔFsBmh1 and ΔFsBmh2 by homologous recombination (Figures S2 and S3). Intriguingly, the deletion of *FsBmh1* resulted in the elevated expression of *FsBmh2* (4.5-fold higher), and the deletion of *FsBmh2* resulted in the elevated expression of *FsBmh1* (2.2-fold higher). However, the reintroduction of a wild copy of *FsBmh1* or *FsBmh2* into the corresponding mutants (C-ΔFsBmh1 and C-ΔFsBmh2) abolished this expression compensation (Figure 2).

### 3.4. Diverse Contributions of FsBmh1 and FsBmh2 to Fungal Phenotypes

**Figure 3:**
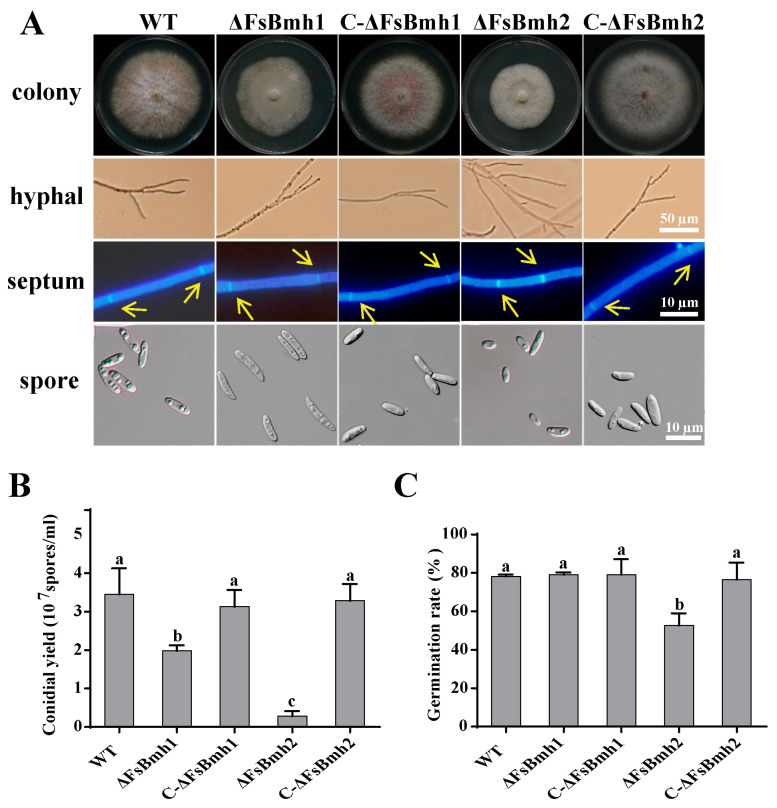
Phenotypes of *FsBmh*-deletion mutants and complementation mutants. (**A**) Hyphal and conidial morphology. Colonies were photographed on PDA plates on day 7 after inoculation. Hyphal branches were photographed on PDA plates on day 3 after inoculation using a microscope, scale bar = 50 µm. To visualize septa, hyphae were stained with CFW and examined under a fluorescent microscope, scale bar = 10 µm. To minimize the error of comparing cells of different ages, only the fourth cells from the tip of a hypha were measured. The septa were marked by yellow arrows. Spore characteristics were photographed by a differential-interference contrast microscope, scale bar = 10 µm. (**B**) Statistics of conidial yield of strains. Conidia were harvested from colonies of 7-days-old PDA plates. (**C**) Conidial germination rate in PDB at 28 °C with rotation of 150 rpm for 6 h. Different letters indicate significant differences at *p* < 0.05.

Relative to the wild-type strain, the ΔFsBmh1 and ΔFsBmh2 mutants displayed a loss of light brown pigmentation and a reduction in growth by approximately 10% and 30%, respectively. The deletion of *FsBmh1* resulted in longer, thinner microspores, while the deletion of *FsBmh2* led to an increased number of hyphal branches, significantly shorter mycelial cells, and smaller microspores (Figure 3A and Tables S4 and S5). These findings indicate that both *FsBmh1* and *FsBmh2* are involved in regulating fungal cell architecture, albeit in distinct manners within *F. sacchari*.

Microconidia quantification revealed that both *FsBmh1* and *FsBmh2* contribute to sporulation, with *FsBmh2* having a more pronounced impact. For instance, the deletion of *FsBmh1* reduced spore production by 43%, while the deletion of *FsBmh2* led to an 80% reduction (Figure 3B). Furthermore, the ΔFsBmh2 spores exhibited a lower germination rate (67%) compared to the wild-type, whereas ΔFsBmh1 did not significantly affect germination (Figure 3C). The reintroduction of wild-type copies of *FsBmh1* into ΔFsBmh1 (C-ΔFsBmh1) or *FsBmh2* into ΔFsBmh2 (C-ΔFsBmh2) fully restored all the observed defects.

### 3.5. FsBmh1 and FsBmh2 Interactively Regulate Spore Morphology in F. sacchari

**Figure 4:**
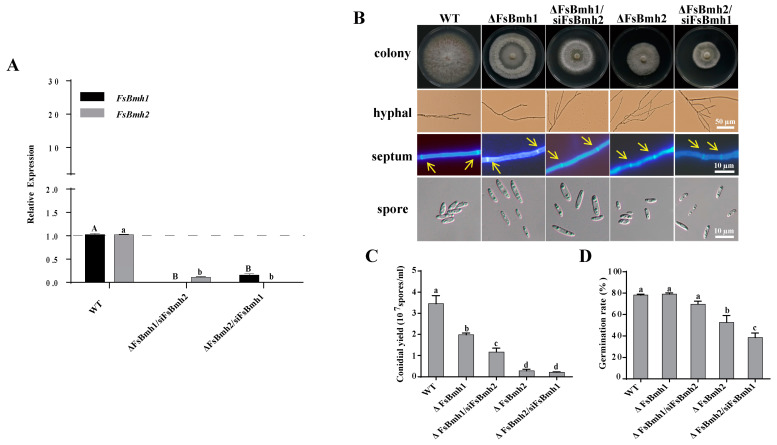
Phenotypes of *FsBmh-*deletion mutants and *FsBmh-*silenced mutants. (**A**) Validation of *FsBmh1* and *FsBmh2* expression using qRT-PCR in ΔFsBmh1/siFsBmh2 and ΔFsBmh2/siFsBmh1. The expression of *FsBmh1* and *FsBmh2* transcripts was measured by quantitative real-time RT-PCR (2^−ΔΔCT^ method) with 18S rRNA as internal reference. The transcript levels of WT were set to a value of 1.0 and indicated by the dotted line. (**B**) Hyphal and conidial morphology. Colonies were photographed on PDA plates on day 7 after inoculation. Hyphal branches were photographed on PDA plates on day 3 after inoculation using a microscope, scale bar = 50 µm. To visualize septa, hyphae were stained with CFW and examined under a fluorescent microscope, scale bar = 10 µm. To minimize the error of comparing cells of different ages, only the fourth cells from the tip of a hypha were measured. The septa were marked by yellow arrows. Spores were photographed by a differential-interference contrast microscope, scale bar = 10 µm. (**C**) Statistics of conidial yield of strains. Conidia were harvested from colonies of 7-days-old PDA plates. (**D**) Conidial germination rate in PDB at 28 °C with rotation of 150 rpm for 6 h. Different letters indicate significant differences at *p* < 0.05.

Attempts to simultaneously knockout both *FsBmh1* and *FsBmh2* in a single strain were unsuccessful, suggesting that the simultaneous deletion of these genes may result in a lethal phenotype in *F. sacchari*. Alternatively, we constructed strains with silenced *FsBmh2* in the ΔFsBmh1 background (ΔFsBmh1/siFsBmh2) and silenced *FsBmh1* in the ΔFsBmh2 background (ΔFsBmh2/siFsBmh1), with expression levels reduced to 15.3% and 11.1% of the wild-type strain, respectively (Figure 4A). At the colony level, silencing *FsBmh2* in ΔFsBmh1 further inhibited mycelial growth compared to ΔFsBmh1 alone, which was similar to ΔFsBmh2; however, silencing *FsBmh1* in ΔFsBmh2 did not significantly affect the growth (Figure 4B and Table S4). Examination of the hyphae revealed that the cell length of ΔFsBmh2 and ΔFsBmh1/siFsBmh2 was significantly shorter than that of the wild-type or ΔFsBmh1 (Figure 4B). The deletion of *FsBmh1* and *FsBmh2* reduced the spore yield by approximately 40% and 80%, respectively, indicating that *FsBmh2* plays a more critical role in sporulation. This was further supported by the intermediate spore yield observed in ΔFsBmh1/siFsBmh2 (Figure 4C). Conversely, the deletion of *FsBmh1* did not affect the spore germination rate, but the deletion of *FsBmh2* reduced the germination rate by about 30% (Figure 4D).

**Figure 5:**
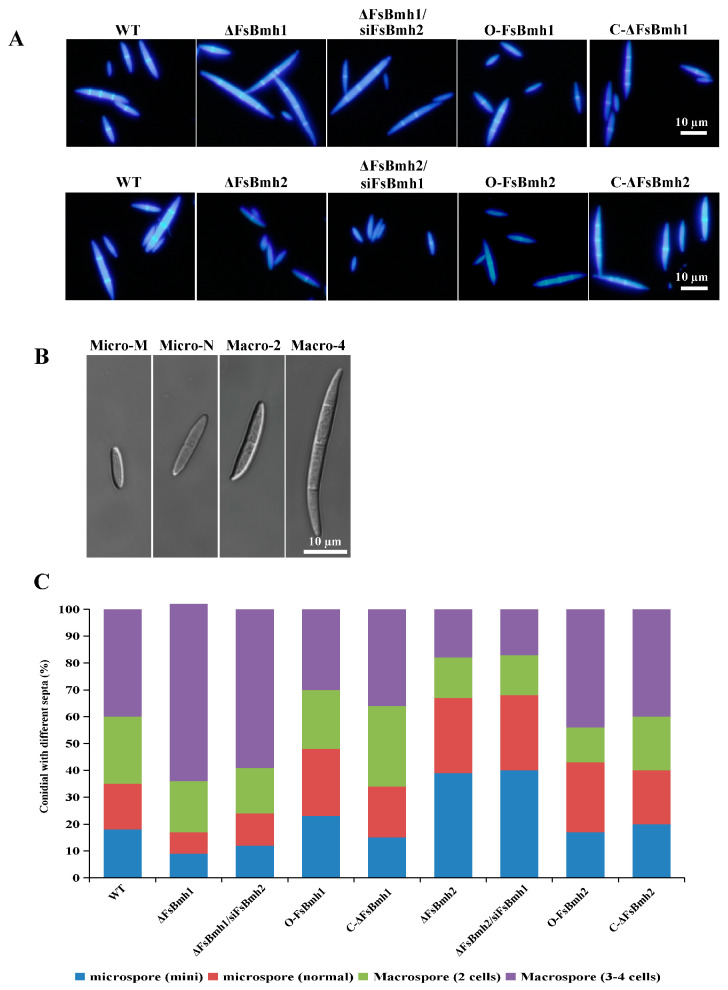
(**A**) Macrospores were induced in carnation leaf medium. Spores were harvested on day 7 and stained by CFW and examined under a fluorescent microscope, scale bar = 10 µm. (**B**) Classification of types of spores. Micro–M = mini microspore; Micro–N = normal microspore; Macro–2 = macrospore with 2 cells; Macro–4 = macrospore with 4 cells, scale bar = 10 µm. (**C**) Distribution of different types of spores (*n* = 100). The images were taken using a differential-interference contrast microscope.

On carnation leaf agar medium, a distribution of 35% microspores (mini and normal) and 65% macrospores (2–4 cells) was observed in the wild-type strain CNO-1. In contrast, the deletion of *FsBmh1* increased the proportion of macrospores to 83%, while the deletion of *FsBmh2* increased the microspores to 67%. The silencing of *FsBmh2* in ΔFsBmh1 completely eliminated the increase in macrospores, whereas the silencing of *FsBmh1* in ΔFsBmh2 did not alter the spore type distribution. The reintroduction of a wild-type copy of *FsBmh1* into ΔFsBmh1 or *FsBmh2* into ΔFsBmh2 fully restored the spore types and proportions. The overexpression of *FsBmh1* or *FsBmh2* did not significantly alter the sporulation pattern (Figure 5).

### 3.6. Overexpression of FsBmh Does Not Alter the Phenotype of F. sacchari

We developed overexpression strains for *FsBmh1* and *FsBmh2*, which exhibited a 20.7-fold and 9.6-fold increase in expression, respectively. No significant phenotypic differences, including hyphal growth, branching, cell length, sporulation, spore morphology, and germination rate, were observed between the overexpression strains (O-*FsBmh*) and the wild-type strain (Figure S6).

### 3.7. FsBmh1 and FsBmh2 Are Essential for F. sacchari Virulence

**Figure 6:**
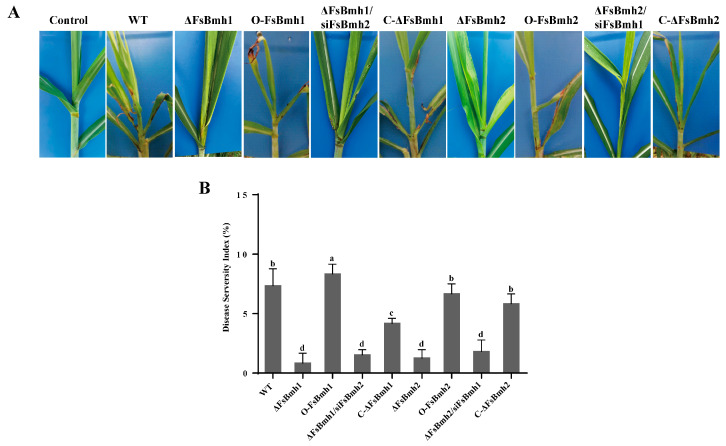
*FsBmh-*deletion mutants are defective in virulence. Each of the plants was injected with 300 µL of spore suspension at concentration of 1 × 10^4^ conidia/mL. (**A**) Symptoms on sugarcane seedlings. Photographs were taken 21 dpi. (**B**) Quantification of disease severity. The disease severity index was determined using 100 seedlings per treatment with 3 replicates. Different letters indicate significant difference at *p* < 0.05.

To assess the impact of *FsBmh1* and *FsBmh2* on the virulence of *F. sacchari*, we inoculated sugarcane plants with conidia from the wild-type and mutant strains. The plants inoculated with the wild-type strain displayed typical PBD symptoms by 14 days post-inoculation (dpi), whereas those inoculated with ΔFsBmh1, ΔFsBmh2, ΔFsBmh1/siFsBmh2, or ΔFsBmh2/siFsBmh1 exhibited significantly milder symptoms by 21 dpi. The virulence of *FsBmh2* was fully restored by reintroducing a wild-type copy, while only about 60% of the virulence was restored in the *FsBmh1* deletion mutant. However, the overexpression of *FsBmh1* resulted in a significant increase in virulence, whereas the overexpression of *FsBmh2* did not (Figure 6).

### 3.8. Transcriptomic Analysis Reveals the Functional Basis of FsBmh1 and FsBmh2

**Figure 7:**
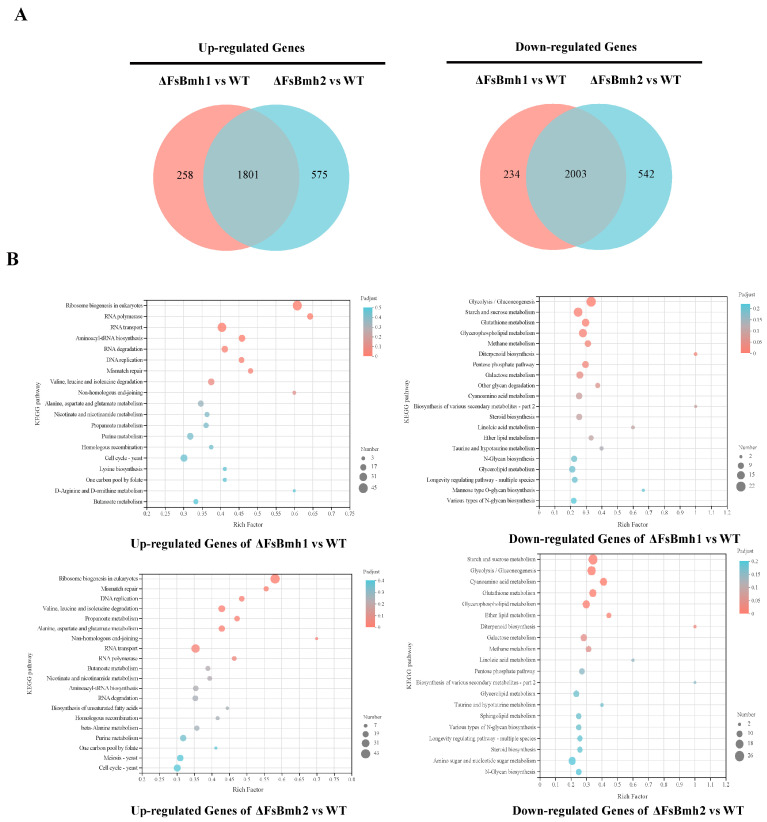
Distribution of DEGs (≥two-fold) in ΔFsBmh1 and ΔFsBmh2 versus WT. (**A**) Venn diagrams showing the overlapped counts of the genes up-regulated or down-regulated in both ΔFsBmh1 and ΔFsBmh2 versus WT. (**B**) KEGG pathway enrichment of genes up-regulated or down-regulated in ΔFsBmh1 and ΔFsBmh2 versus WT.

To understand the mechanisms by which *FsBmh1* and *FsBmh2* regulate fungal phenotypes, we performed comparative transcriptomic analysis on ΔFsBmh1, ΔFsBmh2, and the wild-type (WT) strain. A total of 4296 and 4921 differentially expressed genes (DEGs) were identified between ΔFsBmh1 and WT and between ΔFsBmh2 and WT, respectively. Among these, 3804 DEGs were shared by both mutants (Figure 7A). Kyoto Encyclopedia of Genes and Genomes (KEGG) enrichment analysis indicated that the up-regulated genes in ΔFsBmh1 and ΔFsBmh2 were primarily enriched in pathways related to ribosome biogenesis, RNA polymerase, RNA transport, mismatch repair, and DNA replication. Specifically, the up-regulated DEGs in ΔFsBmh2 were enriched in amino acid and pantothenate metabolism pathways. Conversely, the down-regulated DEGs in both mutants were mainly involved in carbohydrate, amino acid, lipid, and energy metabolism (Figure 7B). Quantitative real-time PCR (qRT-PCR) validation of 15 randomly selected DEGs confirmed the consistency with the transcriptomic data (Figure S7 and Table S2).

**Figure 8:**
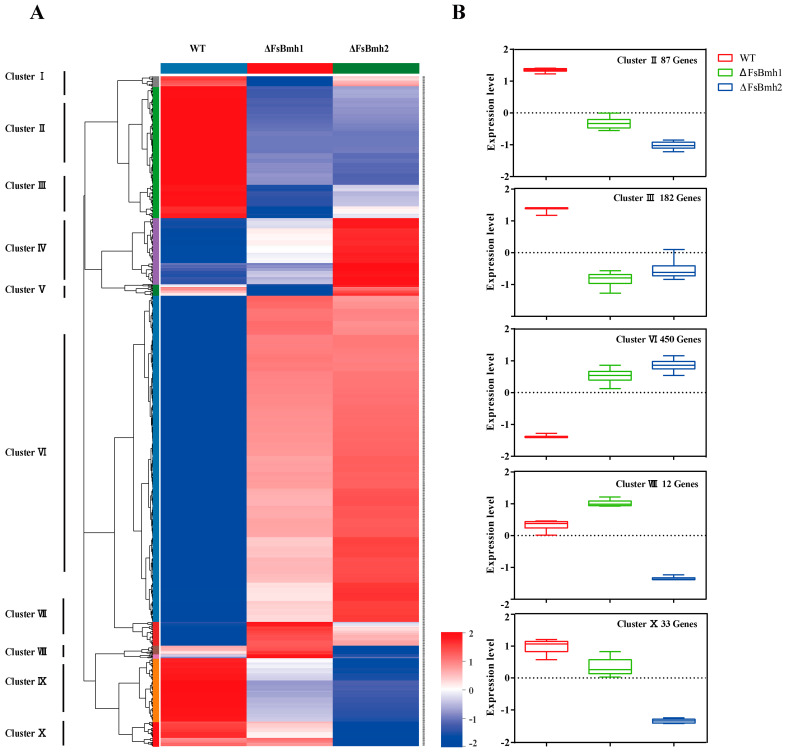
Hierarchical clustering of DEGs among WT, ΔFsBmh1, and ΔFsBmh2. (**A**) A total of 981 DEGs that were enriched in KEGG pathways (ΔFsBmh1 and ΔFsBmh2 versus WT) were clustered into 10 clusters. Red indicates high expression, blue indicates low expression. (**B**) Gene expression values on selected clusters. The dotted line indicates the average expression level of all DEGs within the cluster and its value was set as 0. Color scale shows the level of gene expression of log10^(FPKM+ 1)^.

Hierarchical clustering analysis of DEGs enriched in KEGG pathways identified 981 genes (Table S11) that could be grouped into 10 clusters (Figure 8A). These genes encode proteins crucial for various cellular processes. Cluster VI, with the highest number of genes (450), contained DEGs up-regulated in both ΔFsBmh1 and ΔFsBmh2, with ΔFsBmh2 showing higher expression levels. Most of these genes were involved in DNA repair, mismatch repair, DNA metabolic processes, and stress response, as indicated by gene ontology (GO) enrichment. Notably, these included genes encoding heat shock protein SSB1 (FVER_06187), cell division control protein 45 (FVER_01583), and DNA mismatch repair protein MSH3 (FVER_14666), which align with the stressed phenotypes observed in ΔFsBmh2. Cluster V, consisting of 16 genes, were primarily involved in cysteine and methionine metabolism, with expression most suppressed in ΔFsBmh1 compared to WT and ΔFsBmh1. Cluster X, containing 33 genes with catalytic activity, such as chitin synthase (FVER_07617), gamma-glutamyl transpeptidase (FVER_11123), and malate dehydrogenase (FVER_13093), was specifically down-regulated in ΔFsBmh2. Clusters I through III, comprising 14, 87, and 182 genes, respectively, contained genes that were highly expressed in the WT, including FVER_14638 (1,3-beta-glucosidase), pyruvate kinase (FVER_02565), low-affinity hexose transporter HXT3 (FVER_03150), vesicle-associated membrane protein 7 (Vam7, FVER_01791), and protein transport membrane glycoprotein Sec20 (FVER_03801) (Table S11).

### 3.9. Identification of Direct Targets of FsBmh1 and FsBmh2

Given the modulatory role of 14-3-3 proteins in cellular processes through their interactions with target proteins, we utilized the yeast two-hybrid (Y2H) system to identify the direct interactors of FsBmh1 and FsBmh2. Our analysis revealed a total of 15 and 17 proteins interacting with FsBmh1 and FsBmh2, respectively. Among these, 12 proteins were common targets for both FsBmh1 and FsBmh2, while 3 were specific to FsBmh1 and 5 to FsBmh2. The shared targets are predominantly involved in primary metabolic pathways, including starch and sucrose metabolism, glycolysis, protein and nucleotide synthesis, and energy generation, which are crucial for fungal growth and development. The FsBmh1 or FsBmh2 specific target proteins are implicated in processes such as glycolysis, the tricarboxylic acid cycle, oxidoreduction, meiosis, and cell membrane stability (Table 1). Notably, FsBmh2 was found to interact with a hsp70-like molecular chaperone and the meiosis-related protein Mei5. If the functionality of these proteins is contingent upon their interaction with FsBmh2, the deletion of *FsBmh2* could potentially have a profound effect on the growth and developmental processes of the fungus.

## 4. Discussion

This study elucidates the pivotal roles of the 14-3-3 protein genes *FsBmh1* and *FsBmh2* in the development, growth, conidiation, and virulence of *F. sacchari*, the causative agent of sugarcane Pokkah boeng disease. The observed developmental defects, including pigment loss, growth reduction, and attenuated virulence in the deletion mutants, align with previous findings that 14-3-3 proteins are integral to the regulation of cellular processes in various fungi, such as vegetative development, metabolism, stress response, signaling pathways, transcription factor activity, and pathogenicity [34,35,36,37,38].

The redundancy of essential genes, often present in multiple copies within a genome, ensures functional redundancy [39]. Our findings indicate that while *FsBmh1* or *FsBmh2* can be singly dispensable for growth, their combined deletion is lethal, underscoring their essential roles in *F. sacchari*. The mutual compensation at the transcriptional level observed for *FsBmh1* and *FsBmh2* (Figure 2 and Figure S6A) is consistent with findings in *Beauveria bassiana* and *Ganoderma lucidum* [40,41].

In *F. graminearum, FgBmh1* and *FgBmh2* co-regulate nitrogen sensing, but *FgBmh2* is uniquely required for conidiation and virulence. In contrast, *FsBmh1* and *FsBmh2* exhibit redundant roles in growth, conidiation, and pathogenicity, yet they diverge in the regulation of other phenotypic traits. For instance, the knockdown of *FsBmh2* alone leads to reduced spore germination, shorter hyphal cells, and increased branching (Figure 3 and Figure 4). This contrasts with *B. bassiana*, where conidial germination is accelerated in both 14-3-3 genes deletion mutants, and *G. lucidum*, where silencing either *GlBmh1* or *GlBmh2* results in increased hyphal branching. Notably, *FsBmh1* and *FsBmh2* differentially regulate spore morphology; *FsBmh1* knockdown results in slender spores, while *FsBmh2* knockdown produces thicker, shorter spores under nutrient-rich conditions (Figure 3 and Figure 4). However, on carnation leaf agar medium, *FsBmh1* knockdown enhances macrospore production (Figure 5), a phenomenon not previously reported in filamentous fungi.

Comparative transcriptome analysis revealed that ΔFsBmh1 and ΔFsBmh2 share up to 70.3% of DEGs, which are associated with multiple phenotypes by regulating similar pathways, such as alanine, aspartate, and glutamate metabolism [42,43,44], glycerophospholipid metabolism [45], and starch and sucrose metabolism pathways [46]. The regulatory capacity of 14-3-3 proteins is further underscored by their ability to specifically bind numerous targets, altering their activity, stability, or localization [47]. Y2H analysis identified 20 proteins interacting with FsBmh1 or FsBmh2 (Table 1), including trehalose, 40S ribosomal protein S25, translational elongation factor EF-1, hsp70-like protein, and H+ ATPase, which have been previously reported as 14-3-3 proteins targets [48,49,50,51]. The majority of targets (12/20) were shared by both FsBmh1 and FsBmh2, highlighting their overlapping functions from the transcriptional landscape to protein interaction. Additionally, the common target arginosuccinate lyase (FVER_01513), enriched in alanine, aspartate, and glutamate metabolism, showed up-regulated expression in all the *FsBmh* deletion mutants. In contrast, the transcript levels of trehalase (FVER_04904) and glycerol-3-phosphate acyltransferase (FVER_02466) were down-regulated in the *FsBmh* deletion mutants, with ΔFsBmh1 showing a lesser decrease than ΔFsBmh2 (Figure S7). These findings provide a more comprehensive understanding of the functional overlap between *FsBmh1* and *FsBmh2*.

Furthermore, divergence in functions of *FsBmh1* and *FsBmh2* in germination, hyphal cell length could be linked to clusters of DEGs. For example, *Chs1* (encoding chitin synthase 1) and *BgIB* (β-glucosidase), reported to regulate conidial germination through cell wall remodeling in *Aspergillus fumigatus* and *A. flavus* [52,53], were uniquely decreased in ΔFsBmh2; specific downregulation of *Mae1*, encoding a malate dehydrogenase which has been shown to regulate hyphal septa, cell length, and the number of nuclei in *Arthrobotrys oligospora* [54], was found only in ΔFsBmh2 (Figure 8, cluster X). On the other hand, decrease in aerial hyphae in ΔFsBmh1 was accompanied by the down regulation of expression of *Mon1*, *vps39*, and *Met17*, (Figure 8, cluster V), as these genes have been shown to be critical for aerial hyphae formation and virulence in *F. graminearum* and *Alternaria alternata* [55,56]. Since both FsBmh1 and FsBmh2 locate in the cytoplasm and most of their potential targets also locate in the cytoplasm, these may form a spatial basis for their diversified functions. The discrepancy in target specificity between FsBmh1 and FsBmh2 may constitute the molecular basis for the non-redundant functions of the two 14-3-3 proteins in *F. sacchari*. Notably, Ecm33, a GPI-anchored cell wall organization protein crucial for pathogenicity [57,58], interacts exclusively with FsBmh1, suggesting a potential role for FsBmh1 in modulating host immunity by regulating Ecm33. Conversely, trehalose-6-phosphate phosphatase (Tps2), required for vegetative growth, conidiogenesis, and pathogenicity by maintaining cellular trehalose-6-phosphate balance [59,60,61], specifically interacts with FsBmh2 (Table 1).

In summary, our findings provide novel insights into the roles of *FsBmh1* and *FsBmh2* in *F. sacchari*, and we identified potential targets for PBD control through the manipulation of FsBmh-target interactions, e.g., blocking the active sites of the FsBmhs with small molecules to interfere with their interaction with crucial targets, e.g., Ecm33 and Tps2. In this regard, low homology between *F. sacchari* FsBmhs and sugarcane 14-3-3 proteins (Figure S10) is promising for the development of a safe anti-PBD potent fungicide.

## Figures and Tables

**Table 1:** Binding targets of FsBmh in *F. sacchari*.

No.	Protein ID	Reference	Targeted by	Functional Annotation	Identity/%	E-Value
1	FVER_00270	XP_031079814.1	FsBmh1 & FsBmh2	alpha-glucosidase (maltase)	94.44	0
2	FVER_01513	KAF5563502.1	FsBmh1 & FsBmh2	Arginosuccinate lyase	98.72	0
3	FVER_02281	XP_018749177.1	FsBmh1 & FsBmh2	40S ribosomal protein S25	100	0
4	FVER_02466	KAG5750504.1	FsBmh1 & FsBmh2	glycerol-3-phosphate O-acyltransferase	98.83	0
5	FVER_03743	KAG7434925.1	FsBmh1 & FsBmh2	Glyceraldehyde-3phosphate dehydrogenase	99.41	0
6	FVER_04097	AMD38891.1	FsBmh1 & FsBmh2	Translation elongation factor 1-alpha	100	0
7	FVER_04309	KAI1018791.1	FsBmh1 & FsBmh2	Glutamate decarboxylase	99.48	0
8	FVER_04904	XP_023433270.1	FsBmh1 & FsBmh2	Trehalase	99.13	0
9	FVER_06138	KAI1031434.1	FsBmh1 & FsBmh2	Bifunctional purine biosynthesis protein	99.05	0
10	FVER_06338	XP_023428086.1	FsBmh1 & FsBmh2	F-type H^+^-transporting ATPase subunit gamma	99.67	0
11	FVER_07898	AMD38871.1	FsBmh1 & FsBmh2	Alpha, alpha-trehalose-phosphate synthase	100	0
12	FVER_12178	XP_041687834.1	FsBmh1 & FsBmh2	Phosphotyrosine-specific protein phosphatase	99.27	0
13	FVER_09248	XP_023430651.1	FsBmh1	GPI-anchored cell wall organization protein	98.74	0
14	FVER_10685	KAF5704680.1	FsBmh1	S-(hydroxymethyl) glutathione dehydrogenase	99.48	0
15	FVER_13904	KAF5653012.1	FsBmh1	Pyruvate decarboxylase	99.65	0
16	FVER_02283	KAF5614968.1	FsBmh2	hsp70-like protein	85.99	0
17	FVER_05845	XP_023435700.1	FsBmh2	uricase	100	0
18	FVER_08037	XP_023423671.1	FsBmh2	meiosis 5 (Mei5) protein	94.78	0
19	FVER_11800	KAI1029965.1	FsBmh2	related to helix-loop-helix protein	96.48	0
20	FVER_11869	KAI1041413.1	FsBmh2	trehalose-6-phosphate phosphatase	99.77	0

## Data Availability

Data are contained within the article.

## Supplementary Materials

The following supporting information can be downloaded at: https://www.mdpi.com/article/10.3390/jof10040257/s1, Figure S1: Sequence alignments and analysis of the phylogenetic relationships among 14-3-3 and its homologs; Figure S2: Generation of *FsBmh1* deletion mutant strain; Figure S3: Generation of *FsBmh2* deletion mutant strain; Figure S4: Generation of *FsBmh*-deletion/silenced mutants; Figure S5: Generation of *FsBmh*- overexpression mutants; Figure S6: *FsBmh-*expression and phenotypes of *FsBmh-*overexpression mutants.; Figure S7: RT-qPCR validation of DEGs in ΔFsBmh1 and ΔFsBmh2; Figure S8: The interaction relationship between 14-3-3 proteins and their targets; Figure S9: Cellular localization of 14-3-3 proteins in *F.sacchari*; Figure S10: Sequence alignments and analysis of the phylogenetic relationships between *F. sacchari* FsBmhs and sugarcane 14-3-3 proteins. Table S1: Primer pairs used for the manipulation of *FsBmh1* and *FsBmh2* in *F. sacchari* and the identification of their mutants; Table S2: Primer pairs used in qRT-PCR for assessing transcript levels of 15 DEGs; Table S3: Grading standard of Pokkah boeng disease of sugarcane; Table S4: Effect of *FsBmh* mutation or overexpression on the cell length of the hyphae; Table S5: Effect of *FsBmh* mutation or overexpression on the microspore morphology grown on PDA; Table S6: Effect of *FsBmh* mutation or overexpression on the spores grown on carnation leaf agar; Table S7: KEGG enrichment analysis of up-regulated DEGs between ΔFsBmh1 and WT; Table S8: KEGG enrichment analysis of down-regulated DEGs between ΔFsBmh1 and WT; Table S9: KEGG enrichment analysis of up-regulated DEGs between ΔFsBmh2 and WT; Table S10: KEGG enrichment analysis of down-regulated DEGs between ΔFsBmh2 and WT; Table S11: List and annotation of DEGs in five representation clusters; Table S12: Primer pairs used in Y2H.
