# Supplementary material for: Redundant and Distinct Roles of Two 14-3-3 Proteins in Fusarium sacchari, Pathogen of Sugarcane Pokkah Boeng Disease
Source: J Fungi (Basel). 2024 Mar 28;10(4):257. doi: 10.3390/jof10040257 (PMC11051555; doi:10.3390/jof10040257)
Supplement: Supplementary file 1 [file jof-10-00257-s001.zip › Figure S1.pdf]

A

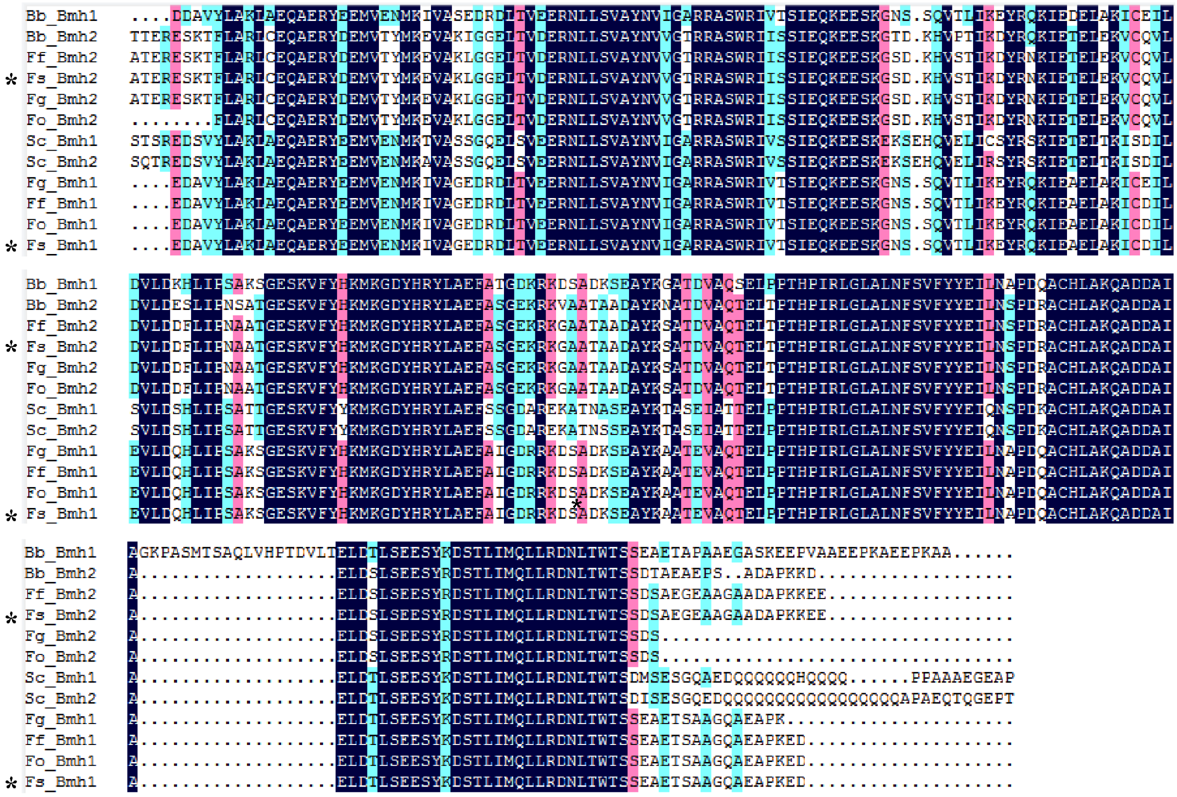

B

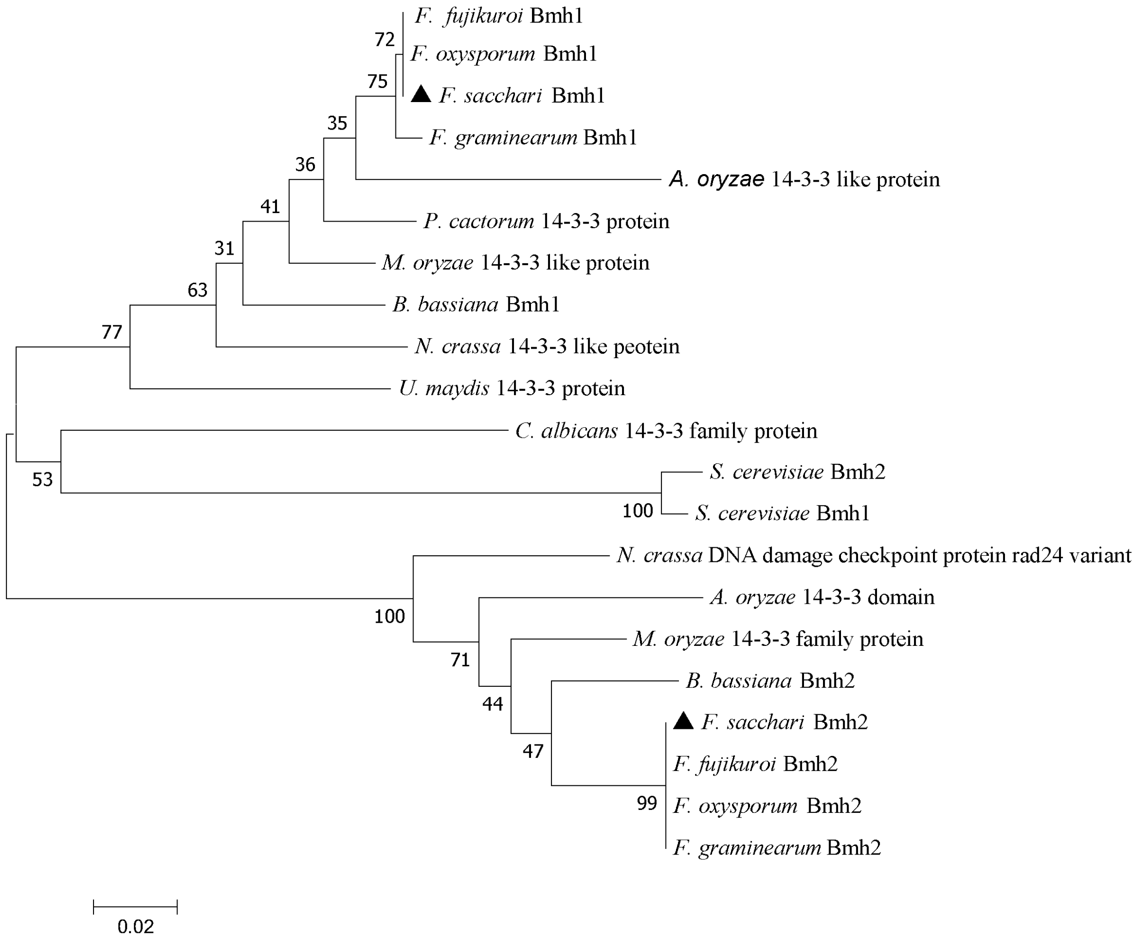

Figure S1. Sequence alignments and analysis of the phylogenetic relationships among 14-3-3 and its homologs. (A) sequence alignment of 14-3-3 and its homologs. (B) A neighbor-joining tree was constructed using the amino acid sequence of 14-3-3 proteins from different organisms.
