# Supplementary material for: Redundant and Distinct Roles of Two 14-3-3 Proteins in Fusarium sacchari, Pathogen of Sugarcane Pokkah Boeng Disease
Source: J Fungi (Basel). 2024 Mar 28;10(4):257. doi: 10.3390/jof10040257 (PMC11051555; doi:10.3390/jof10040257)
Supplement: Supplementary file 1 [file jof-10-00257-s001.zip › Figure S4.pdf]

**A**

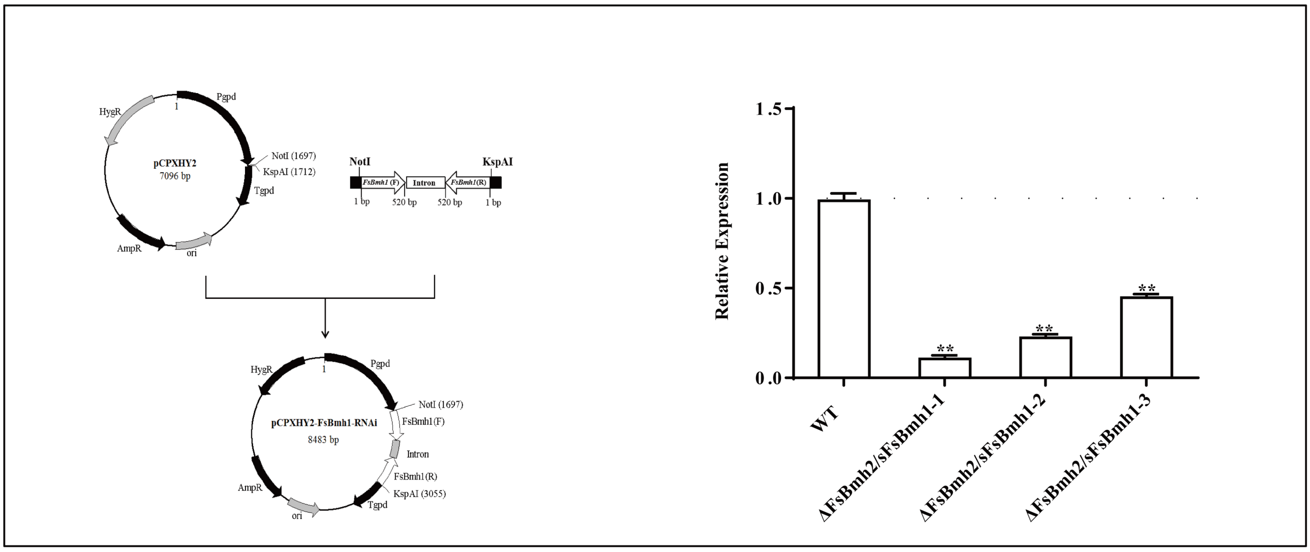


**B**

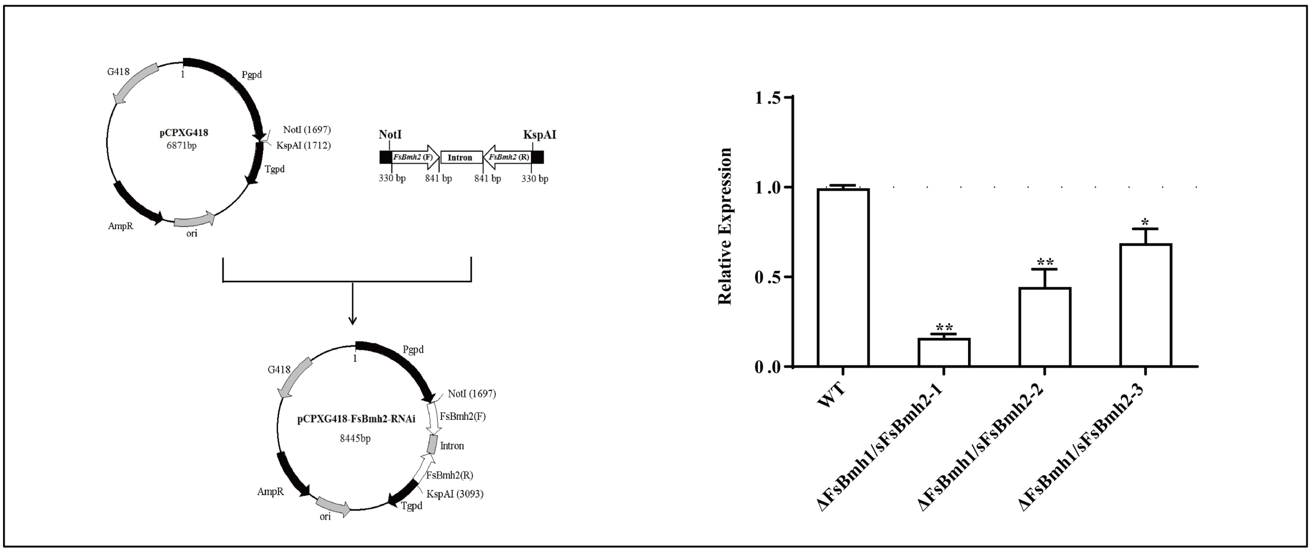


**Figure S4**. Generation of FsBmh-deletion/silenced mutants. (A,B) Silenced *FsBmh1* in ΔFsBmh2 and silenced *FsBmh2* in ΔFsBmh1. FsBmh- RNA interference vector was generated by insertion of the *FsBmh*-RNAi cassette using in-fusion strategy (see Materials and Methods). The mean and standard deviation were derived from three independent biological replicates, each of which contained three technical repeats. The expression of *FsBmh*- transcripts was measured by quantitative real-time RT-PCR(2−ΔΔCT method). P-value were calculated based on Student's T-test of replicate $2^{-\Delta\Delta CT}$ values between a sample and WT. **P* <0.05, ***P* <0.01, ****P* <0.001.
