# Supplementary material for: Redundant and Distinct Roles of Two 14-3-3 Proteins in Fusarium sacchari, Pathogen of Sugarcane Pokkah Boeng Disease
Source: J Fungi (Basel). 2024 Mar 28;10(4):257. doi: 10.3390/jof10040257 (PMC11051555; doi:10.3390/jof10040257)
Supplement: Supplementary file 1 [file jof-10-00257-s001.zip › Figure S5.pdf]

A

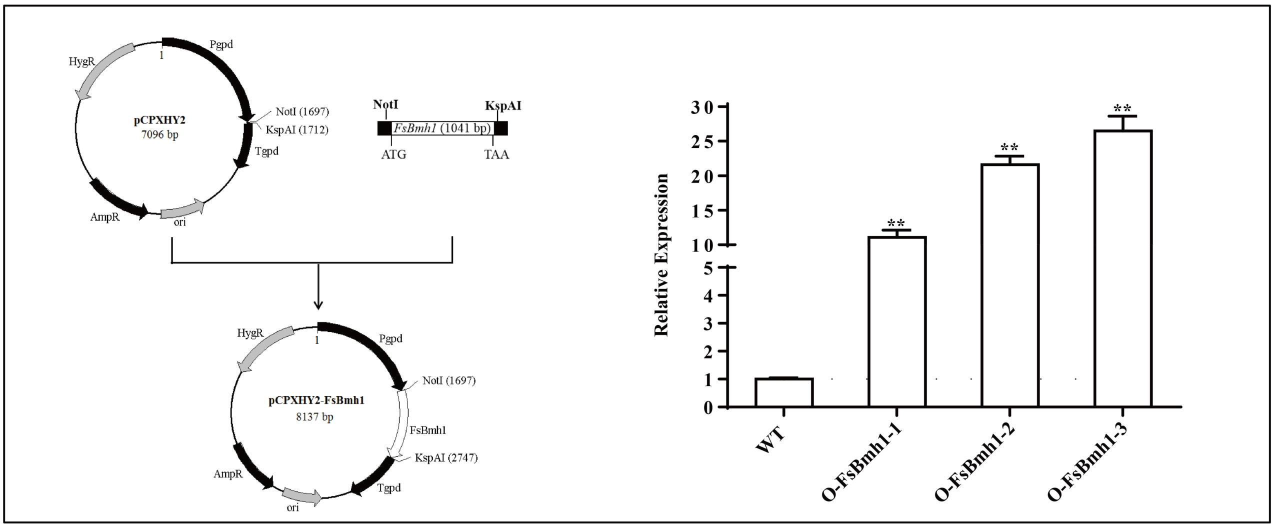

B

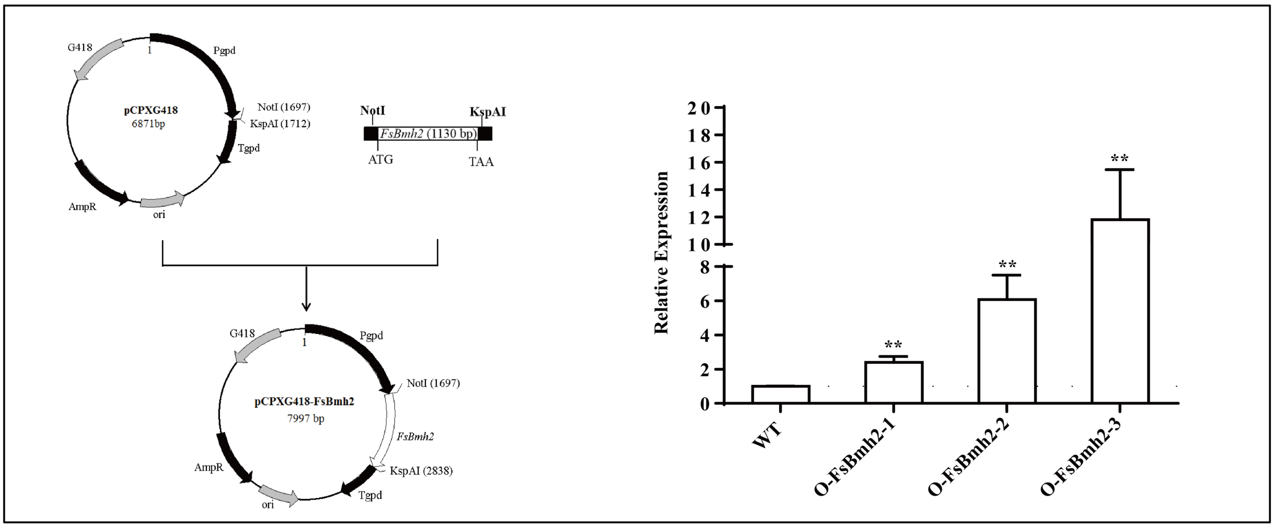

Figure S5. Generation of *FsBmh-* overexpression mutants. (A,B) overexpression *FsBmh1* and *FsBmh2* in wide type. *FsBmh-* overexpression vector was generated by insertion of the *FsBmh-* genes using in-fusion strategy (see Materials and Methods). *P*-value were calculated based on Student's T-test of replicate $2^{-\Delta\Delta CT}$ values between a sample and WT. **P*<0.05, ***P*<0.01, ****P*<0.001.
