# Supplementary material for: Redundant and Distinct Roles of Two 14-3-3 Proteins in Fusarium sacchari, Pathogen of Sugarcane Pokkah Boeng Disease
Source: J Fungi (Basel). 2024 Mar 28;10(4):257. doi: 10.3390/jof10040257 (PMC11051555; doi:10.3390/jof10040257)
Supplement: Supplementary file 1 [file jof-10-00257-s001.zip › Figure S6.pdf]

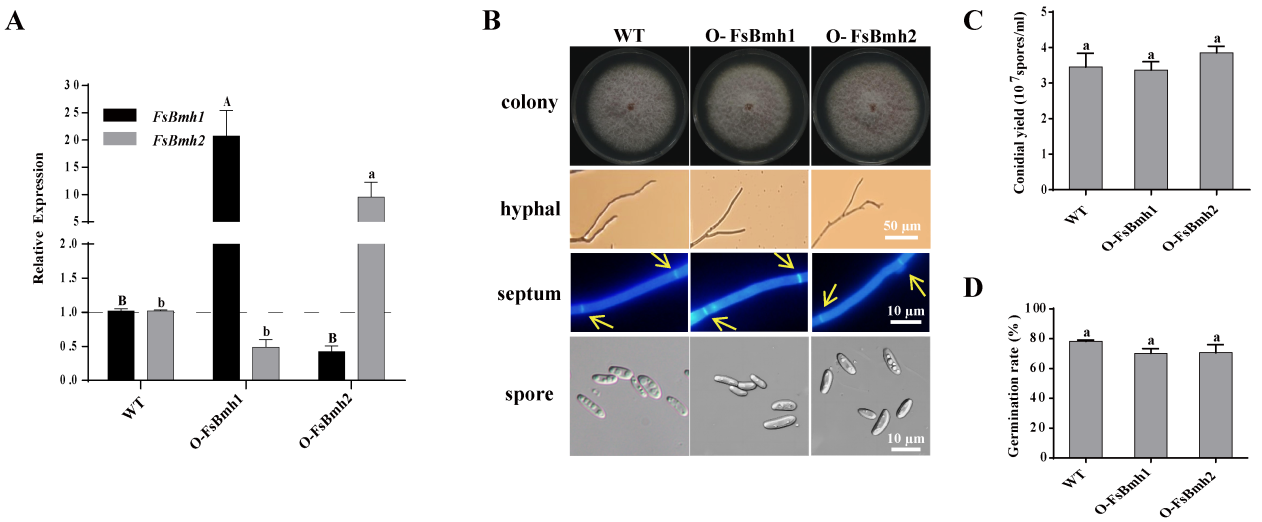

Figure S6. *FsBmh-* expression and phenotypes of *FsBmh-* overexpression mutants. (A) Relative expression of *FsBmh-* genes in FsBmh-overexpression mutants were determined by qRT-PCR. (B) Hyphal and conidial morphology. Colony were photographed on PDA plates at day 7 after inoculation. Hyphae of 3 day old were photographed microscopely. To visualize septa, hyphae were stained with CFW. The separations were marked by yellow arrows. Spore characteristics were photographed by microscope, scale bar = 10 µm. (C) Statistics of conidial yield of strains. Conidia were harvested from colonies of 7-day-old PDA plates (D) Conidial germination rate in PDB at 28℃ with rotation of 150 rpm for 6 h. Different letters indicate significant differences at $P < 0.05$.
