# Supplementary material for: Redundant and Distinct Roles of Two 14-3-3 Proteins in Fusarium sacchari, Pathogen of Sugarcane Pokkah Boeng Disease
Source: J Fungi (Basel). 2024 Mar 28;10(4):257. doi: 10.3390/jof10040257 (PMC11051555; doi:10.3390/jof10040257)
Supplement: Supplementary file 1 [file jof-10-00257-s001.zip › Figure S7.pdf]

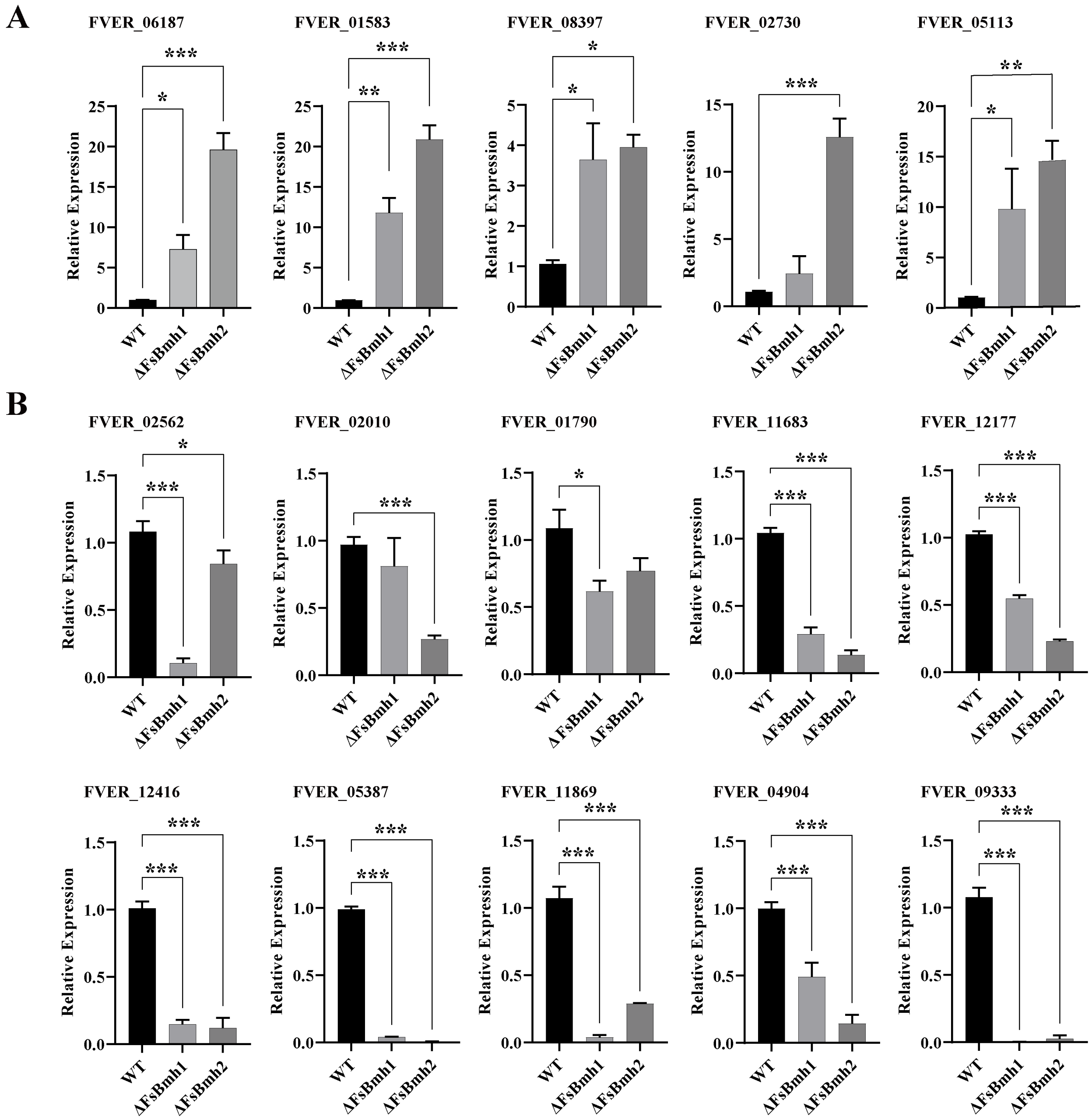

Figure S7. Validation of DEGs in ΔFsBmh1 and ΔFsBmh2. (A) Up-regulated genes in ΔFsBmh1 and ΔFsBmh2 versus WT. (B) Down-regulated genes in ΔFsBmh1 and ΔFsBmh2 versus WT. P-value were calculated based on Student's T-test of replicate $2^{-\Delta\Delta CT}$ values between a sample and WT. **P* < 0.05, ***P* < 0.01, ****P* < 0.001.
