# Supplementary material for: Redundant and Distinct Roles of Two 14-3-3 Proteins in Fusarium sacchari, Pathogen of Sugarcane Pokkah Boeng Disease
Source: J Fungi (Basel). 2024 Mar 28;10(4):257. doi: 10.3390/jof10040257 (PMC11051555; doi:10.3390/jof10040257)
Supplement: Supplementary file 1 [file jof-10-00257-s001.zip › Figure S8.pdf]

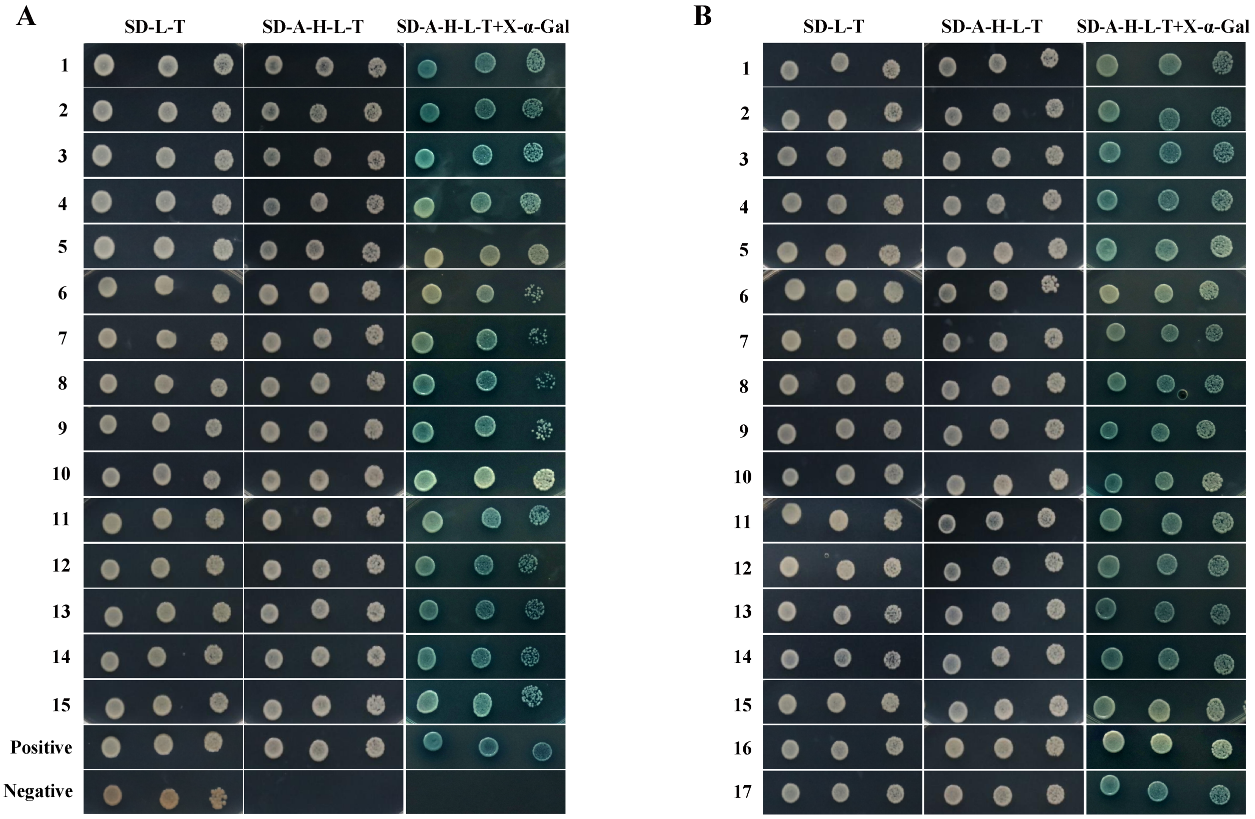

Figure S8. The interaction relationship between 14-3-3 proteins and their targets. (A) Yeast two hybird assay for the interaction relationship of FsBmh1. (B) Yeast two hybird assay for the interaction relationship of FsBmh2.
