# Supplementary material for: Redundant and Distinct Roles of Two 14-3-3 Proteins in Fusarium sacchari, Pathogen of Sugarcane Pokkah Boeng Disease
Source: J Fungi (Basel). 2024 Mar 28;10(4):257. doi: 10.3390/jof10040257 (PMC11051555; doi:10.3390/jof10040257)
Supplement: Supplementary file 1 [file jof-10-00257-s001.zip › Figures S2-S3.pdf]

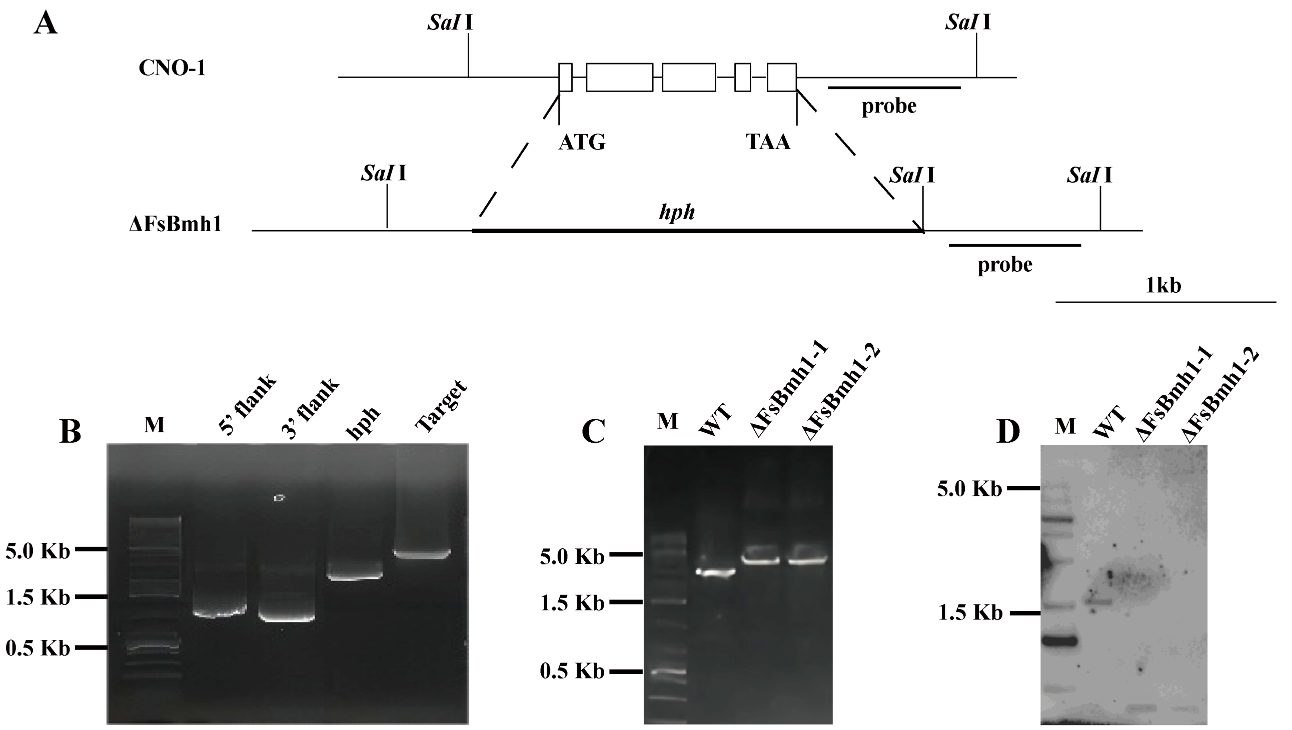

Figure S2. Generation of FsBmh1 deletion mutants strains. (A,B) *FsBmh1* gene locus and gene replacement construct. (C) *FsBmh1* deletion mutants were validated by PCR with FsBmh1-A/B. (D) FsBmh1 deletion mutants strains were validated by Southern Blot. The probes have been marked in the Figure.

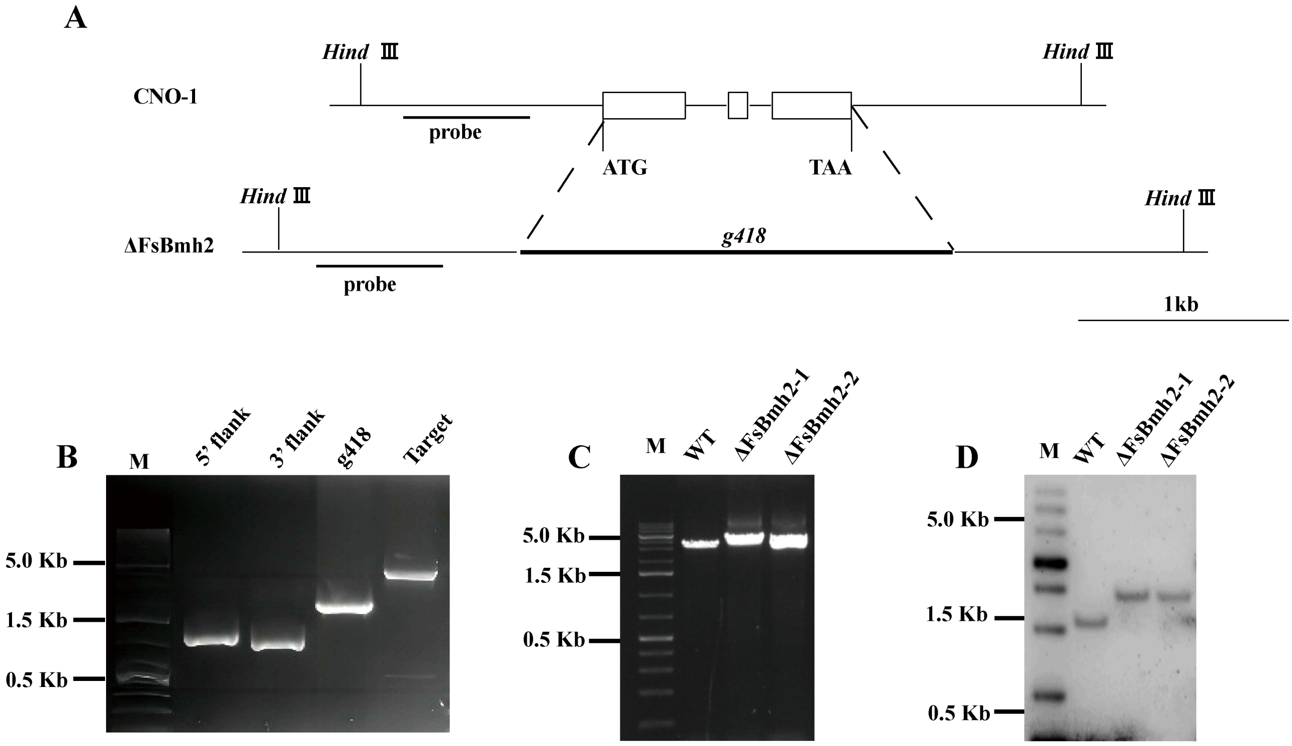

Figure S3. Generation of FsBmh2 deletion mutants strains. (A,B) *FsBmh2* gene locus and gene replacement construct. (C) FsBmh2 deletion mutants were validated by PCR with FsBmh2-A/B. (D) *FsBmh2* deletion mutants strains were validated by Southern Blot. The probes have been marked in the Figure.
