# Supplementary material for: Redundant and Distinct Roles of Two 14-3-3 Proteins in Fusarium sacchari, Pathogen of Sugarcane Pokkah Boeng Disease
Source: J Fungi (Basel). 2024 Mar 28;10(4):257. doi: 10.3390/jof10040257 (PMC11051555; doi:10.3390/jof10040257)
Supplement: Supplementary file 1 [file jof-10-00257-s001.zip › Figures S9-S10.pdf]

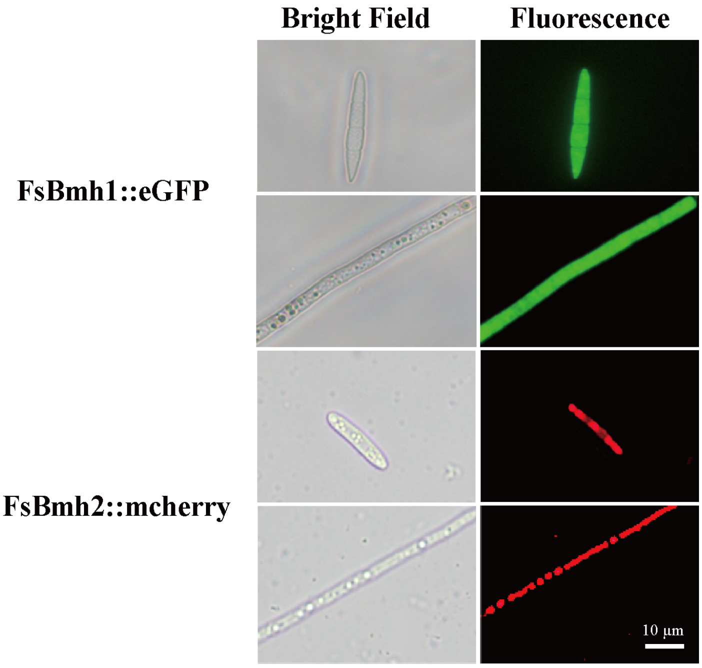

Figure S9. Cellular localization of 14-3-3 proteins in *F. sacchari*.

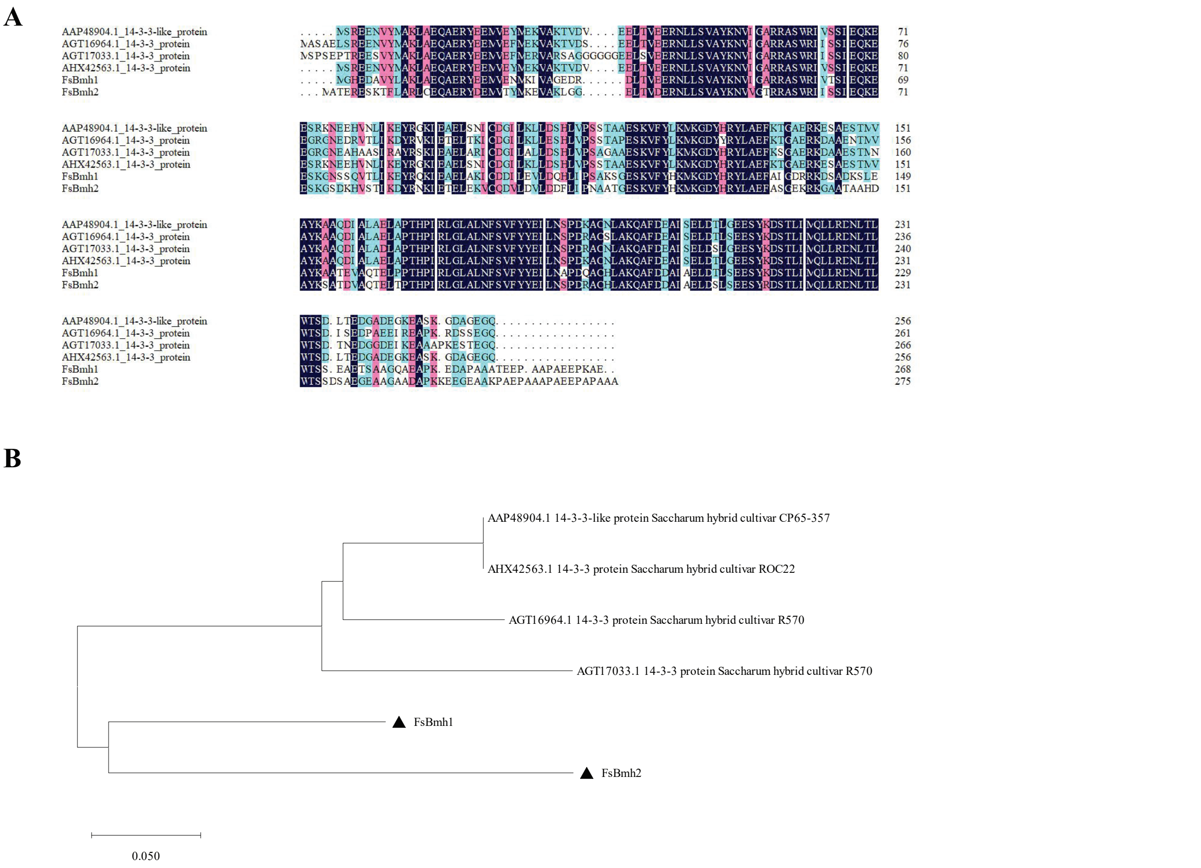

Figure S10. Sequence alignments and analysis of the phylogenetic relationships between *F. sacchari* FsBmhs and Sugarcane 14-3-3 proteins.
