# Supplementary material for: Redundant and Distinct Roles of Two 14-3-3 Proteins in Fusarium sacchari, Pathogen of Sugarcane Pokkah Boeng Disease
Source: J Fungi (Basel). 2024 Mar 28;10(4):257. doi: 10.3390/jof10040257 (PMC11051555; doi:10.3390/jof10040257)
Supplement: Supplementary file 1 [file jof-10-00257-s001.zip › Table S1.pdf]

Table S1. Primer pares used for the manipulation of *FsBmh1* and *FsBmh2* in *F. sacchari* and the identification of their mutants.

Primers	Sequence(5'-3')	Purpose
FsBmh1-A/B	GTGAAGAGCGATTGTCCATTGTTGG/CACAGGTCACAGTCACCTCATC	*FsBmh1* full-length clone
FsBmh2-A/B	CACAAGCTCCGCAGTAGGTATCTG/CCAGGTTACTCAATCGTCGCAC	*FsBmh2* full-length clone
FsBmh1-1F/R	TGGTTGATGGCACTGGCAGT/TCTTTCTAGAGGATCCCCGGGTACCGCGACTGTGT TTCAGGACAGT	Cloning of *FsBmh1* 5'
FsBmh2-1F/R	ATGGCACTGTCGGTTGTCTTG/AGTACAATCTGCTCTGATGCCGATTCCTAGACGAG CTGAAC	Cloning of *FsBmh2* 5'
FsBmh1-2F/R	ATATCATCTTCTGTCGACCTGCAGGCTGATTTGTCTATGCAGGTGTC/TCCTCTGTCT GCTTCACAGTC	Cloning of *FsBmh1* 3'
FsBmh2-2F/R	CTTCAATATCATCTTCTGTCGTAGGAACCGAGACGTATTCT/CAGGTACGAGAGACT ATCAC	Cloning of *FsBmh2* 3'
Hph-F/R	CGGTACCCGGGGATCCTCTAG/GCCTGCAGGTCGACAGAAGATG	*Hph* full-length clone
G418- F/R	GGCATCAGAGCAGATTGTACT/CGACAGAAGATGATATTGAAG	*G418* full-length clone
Hph-F0/R0	TGGGCTGATCTGACCAGTTGCC/CACCAGCCCTGGGTTCGCAAAG	Detection of ΔFsBmh1
G418- F0/R0	GGTATGACCGGGTCGTTCAC/ATTCCGTCACCAGCCCTGGG	Detection of ΔFsBmh2
C-FsBmh1-F/R	GAATTCTCTTAGACAACGATGTCAGCAAATC/AGCGTTAACTTCACAGGTCACAGT CACCTCATC	Vector for C-FsBmh1
C-FsBmh2-F/R	GTTAACAAGCTTGCGGCCGCAGAGATTGGCGTCAACTGCC/TCATCTTCTGTCGAC GAATTCTTTGCCACACCTCCATCACC	Vector for C-FsBmh2
s-FsBmh1-1F/R	TTAGAGGTACGCGGCCGCATGGGTCACGAAGACGCCGT/CTTGCTCACCATGCGG CCGGGCAAGACCCAGGCGGATA	*FsBmh1*-sense repeat
s-FsBmh1-2F/R	AGGTCAGCATGCGTTAACATGGGTCACGAAGACGCCGT/GAGCGCACCATCTTCG TTGGGCAAGACCCAGGCGGATA	*FsBmh1*-antisense repeat
s-FsBmh2-1F/R	TTAGAGGTACGCGGCCGCGCCAGGATGTTCTCGATGTC/CTTGCTCACCATGCGGC CTTAGGAGGCAGCAGCAGGCG	*FsBmh2*-sense repeat
s-FsBmh2-2F/R	AGGTCAGCATGCGTTAACGCCAGGATGTTCTCGATGTC/GAGCGCACCATCTTCGT TTTAGGAGGCAGCAGCAGGCG	*FsBmh2*-antisense repeat
O-FsBmh1-F/R	TACTACTTTTAGAGGTACGCATGGGTCACGAAGACGCCGT/AGATCAGGTCAGCAT GCGTTTTACTCGGCCTTAGGCTCCT	Construction of plasmid for O-FsBmh1
O-FsBmh2-F/R	TACTACTTTTAGAGGTACGCATGGTATCGCTGACATCGTA/AGATCAGGTCAGCATG CGTTTTAGGAGGCAGCAGCAGGC	Construction of plasmid for O-FsBmh2
