# Supplementary material for: Redundant and Distinct Roles of Two 14-3-3 Proteins in Fusarium sacchari, Pathogen of Sugarcane Pokkah Boeng Disease
Source: J Fungi (Basel). 2024 Mar 28;10(4):257. doi: 10.3390/jof10040257 (PMC11051555; doi:10.3390/jof10040257)
Supplement: Supplementary file 1 [file jof-10-00257-s001.zip › Table S12.pdf]

Table S12. Primer pares used in Y2H.

No.	Gene ID	Primer sequence (forward/reverse, 5'-3')
1	FVER_00270	GACGTACCAGATTACGCTCATATATGACTCAAGTGCCCGA/TCGATGCCCACCCGGGTGGAATTTCAAACAATACCAAGTA
2	FVER_01513	GACGTACCAGATTACGCTCATATATGGCAGCGAACAAGGC/TCGATGCCCACCCGGGTGGAATTCTAATCCAACATAGCTT
3	FVER_02281	GACGTACCAGATTACGCTCATATATGGCCCCCGCCGCTGG/TCGATGCCCACCCGGGTGGAATTTTAGTCAGAACCACCGACGGC
4	FVER_02466	GACGTACCAGATTACGCTCATATATGTCTCAACATGCTGG/TCGATGCCCACCCGGGTGGAATTTTACGCGTTCTTCTTTC
5	FVER_03743	GACGTACCAGATTACGCTCATATATGGCTCCCATCAAGGTCG/TCGATGCCCACCCGGGTGGAATTTTACTTGGAGGCATCGACCTTGGC
6	FVER_04097	GACGTACCAGATTACGCTCATATATGGGTAAGGAGGACAAG/TCGATGCCCACCCGGGTGGAATTTTATTTCTTGGCGGCCT
7	FVER_04309	GACGTACCAGATTACGCTCATATATGAGTCTCGTACAACATTC/TCGATGCCCACCCGGGTGGAATTCTAGCAAATGGCATGTG
8	FVER_04904	GACGTACCAGATTACGCTCATATAGGAACTATTCTGCTGTG/TCGATGCCCACCCGGGTGGAATTGTTAGAAATGAAACAGA
9	FVER_06138	GACGTACCAGATTACGCTCATATATGAGGCGCCCTCTTCC/TCGATGCCCACCCGGGTGGAATTTTAGTGGTGGAACAGACGAATG
10	FVER_06338	GACGTACCAGATTACGCTCATATATGTTGTCACGAGCCGC/TCGATGCCCACCCGGGTGGAATTTTACATGTCCGCAGAGG
11	FVER_07898	GACGTACCAGATTACGCTCATATATGCCCGGTGCTGTCGATGAGAA/TCGATGCCCACCCGGGTGGAATTTGGCTGCGAATTGGCTTCCT
12	FVER_12178	GACGTACCAGATTACGCTCATATATGAAGACTCCCGCACGA/TCGATGCCCACCCGGGTGGAATTTTAGTGGCGGAGGCTGC
13	FVER_09248	GACGTACCAGATTACGCTCATATATGCATTCCGTTAAGGTCCTCTCAG/TCGATGCCCACCCGGGTGGAATTTTAAAAGAGCTGGGCAACGGC
14	FVER_10685	GACGTACCAGATTACGCTCATATATGTCTACCGTGGGCAAGACC/TCGATGCCCACCCGGGTGGAATTCATATCGACGACGGCACGG
15	FVER_13904	GACGTACCAGATTACGCTCATATATGAGCAGCACCAACCTCCAAAAG/TCGATGCCCACCCGGGTGGAATTTTAGTGCTTCTTGGCATTGTTC
16	FVER_02283	GACGTACCAGATTACGCTCATATATGGCTCCTGCCGTCGGT/TCGATGCCCACCCGGGTGGAATTCCACCAGCTCCGTAGAACTTCA
17	FVER_05845	GACGTACCAGATTACGCTCATATATGCCTTACGTATCTGCTGC/TCGATGCCCACCCGGGTGGAATTTTATAGCTTTGATTGCAAGTTGTC
18	FVER_08037	GACGTACCAGATTACGCTCATATATGCCGTCGCACAACGAAG/TCGATGCCCACCCGGGTGGAATTTTAATTGCCCTTTTCGGTACCAATAC
19	FVER_11800	GACGTACCAGATTACGCTCATATATGGCGGGGGATCATAACG/TCGATGCCCACCCGGGTGGAATTTTAACCTTCTTTCCCATCATCCTC
20	FVER_11869	GACGTACCAGATTACGCTCATATATGGCAACCTCCAGTGA/TCGATGCCCACCCGGGTGGAATTTTACCGCTCATCATTGG
