# Supplementary material for: Redundant and Distinct Roles of Two 14-3-3 Proteins in Fusarium sacchari, Pathogen of Sugarcane Pokkah Boeng Disease
Source: J Fungi (Basel). 2024 Mar 28;10(4):257. doi: 10.3390/jof10040257 (PMC11051555; doi:10.3390/jof10040257)
Supplement: Supplementary file 1 [file jof-10-00257-s001.zip › Table S2.pdf]

Table S2. Primer pares used in qRT-PCR for assessing transcript levels of DEGs.

Gene ID	Anotation	Sequence (forward/reverse, 5'-3')
FVER_02495	18S ribosomal RNA	TGGTTTCTAGGACCGCCGTA/CCTTGGCAAATGCTTTCGC
FVER_02010	Cell division control protein CDC3	TTGTTGGTGAGTCTGGCTTAGGCA/GAATAATGTCGAGGCTGGGTCCC
FVER_02562	Growth-Arrest-Specific Protein	ATGACCGATCTCAACGCCCAAG/CGACAGTAGCAGGACCGAGATTCA
FVER_01790	Peroxisome assembly protein	CTTCGGGGCACCTTTGACGA/TGGTAAGTAGGTAGCGGAGGGTCG
FVER_11683	Outer spore wall protein RRT8	TCTTTAAGGAGGACTATGCCCAGGC/CAAAGTGAGCGATGGGAGATGAAGT
FVER_12177	Phosphatidylserine synthase	CCTCCAACAGCCCAGCAATTG/GAGAGTAATCAAGTCGGCAAGGTGC
FVER_12416	Egh16-like virulence factor	TGGCCTTGGGAGCTGAGCAT/CTCCAGTGGAGTTTTGAATGCCG
FVER_05387	Integral membrane protein PTH11	CCGCCTATGCGACGTTCAAGA/GCAGACTCCAAAGATGAGAACCCAG
FVER_11869	Trehalose-6-phosphate phosphatase	AGGCGGTGATTGGGACTTGAAA/GGTGTGATTCCATGGGGTAGCG
FVER_04904	Trehalase	TTCCGGTCTACATCAAC/CACCGCTATAAACCTCACCAGATGG
FVER_09333	G protein-coupled receptor catalytic subunit	GGTCGTGATGCCTACTA/GGGCGGAGAATGGGTATGCAT
FVER_07211	14-3-3 family protein Bmh1	CGTTCTTGTAGGCGACGGAGAG/TGAGCGATATGAGGAGATGGTTGAG
FVER_01000	14-3-3 family protein Bmh2	TCTCGCTCTCAACTTCTCCGTCTT/AAGTCCAGAGGGTGAGGTTGTCTC
FVER_06187	Heat shock protein SSB1	TTCACTCTTGCCCCTATCCCTCC/CAGAGTTGGAGATAGTGATGTTGGCG
FVER_08397	DNA repair protein RAD50	GGGCGGCGCTTTCATTCAT/GCGGGTGGTCTTCTTGACGGTA
FVER_01583	Cell division control protein 45	CAGAAAATACGAGAGGATAAGCGCG/GCGTCAGAGTCTGAGCTATCGGAGT
FVER_02730	Double-strand break repair protein MRE11	CCTGCACCTGCCAAGAAACCA/GTCTTCGAATGCCTTGCGGC
FVER_05113	Type 2A phosphatase activator TIP41	ACAACGACACACACGCAAAGCAG/GGAATGCCAATCTTTTCGGTGAGT
