# Supplementary material for: Redundant and Distinct Roles of Two 14-3-3 Proteins in Fusarium sacchari, Pathogen of Sugarcane Pokkah Boeng Disease
Source: J Fungi (Basel). 2024 Mar 28;10(4):257. doi: 10.3390/jof10040257 (PMC11051555; doi:10.3390/jof10040257)
Supplement: Supplementary file 1 [file jof-10-00257-s001.zip › Table S3.pdf]

Table S3. Grading scheme for Pokkah boeng disease of sugarcane

Incidence levels	Symptom description
0	No symptom
1	Chlorotic or greensick symptom at the base of young leaves; slight wrinkling; very few irregular reddish specks or stripes
2	The affected leaves are narrower or shorter than the normal leaves; distinct distortion (wrinkling and twisting) and the reddish areas sometimes develop into lens-shaped holes which have no definite arrangement, or reddish tissue may form ladder-like lesions often with dark edges
3	The leaves are affected in severe cases; leaf sheaths become chlorotic and develop irregular necrotic areas of reddish color; the infection in the spindle continues downward into the stalk and dark reddish streaks may be found extending through several internodes; or the infection may form long lesions with cross depressions that give them a ladder-like appearance
4	These lesions sometimes break through the surface of the rind causing curvature and distortion of the stalk. Exaggerated versions of these depressions may look like neatly made ‘‘knife-cuts’’ in the stalk. In the stem, the fungus causes a dark-brown discoloration of the infected tissues. The ladder-like lesions are due to rupturing of the diseased cells which cannot keep up with the growth of the healthy tissue
5	Entire top of the plant dies (referred to as ‘‘top rot’’)
