# Supplementary material for: Redundant and Distinct Roles of Two 14-3-3 Proteins in Fusarium sacchari, Pathogen of Sugarcane Pokkah Boeng Disease
Source: J Fungi (Basel). 2024 Mar 28;10(4):257. doi: 10.3390/jof10040257 (PMC11051555; doi:10.3390/jof10040257)
Supplement: Supplementary file 1 [file jof-10-00257-s001.zip › Table S4.pdf]

Table S4. Effect of *FsBmh* mutation or overexpression on the cell length of the hyphae*

Strain	Distance between two septa (μm)
WT	20.0 ± 3.86[a]
ΔFsBmh1	19.9 ± 0.86[a]
O-FsBmh1	24.5 ± 2.12[a]
ΔFsBmh1/sFsBmh2	12.6 ± 1.08[b]
C-ΔFsBmh1	20.8 ± 3.34[a]
ΔFsBmh2	11.9 ± 1.13[b]
O-FsBmh2	22.1 ± 0.24[a]
ΔFsBmh2/sFsBmh1	9.9 ± 1.03[b]
C-ΔFsBmh2	22.1 ± 2.31[a]

*The wild-type or mutants were grown in PDB for 8 hours. The third cell from the tip of a hypha was measured. Three groups each with 30 hyphae, were determined for cell length between a pair of septa.
