# Supplementary material for: Redundant and Distinct Roles of Two 14-3-3 Proteins in Fusarium sacchari, Pathogen of Sugarcane Pokkah Boeng Disease
Source: J Fungi (Basel). 2024 Mar 28;10(4):257. doi: 10.3390/jof10040257 (PMC11051555; doi:10.3390/jof10040257)
Supplement: Supplementary file 1 [file jof-10-00257-s001.zip › Table S5.pdf]

Table S5. Effect of *FsBmh* mutation or overexpression on the microspore morphology grown on PDA.

Strain	Spore length (μm)	Spore width (μm)
WT	13.63 ± 0.54^{b}	3.38 ± 0.21^{a}
ΔFsBmh1	17.92 ± 1.02^{a}	2.35 ± 0.27^{b}
O-FsBmh1	13.92 ± 1.87^{b}	3.35 ± 0.20^{a}
ΔFsBmh1/sFsBmh2	16.60 ± 1.70^{a}	3.38 ± 0.12^{a}
C-ΔFsBmh1	14.05 ± 0.26^{b}	3.44 ± 0.16^{a}
ΔFsBmh2	10.85 ± 0.70^{c}	3.55 ± 0.15^{a}
O-FsBmh2	13.99 ± 1.26^{b}	3.31 ± 0.17^{a}
ΔFsBmh2/sFsBmh1	10.79 ± 1.26^{c}	2.54 ± 0.38^{b}
C-ΔFsBmh2	14.04 ± 0.61^{b}	3.33 ± 0.14^{a}
