# Supplementary material for: Redundant and Distinct Roles of Two 14-3-3 Proteins in Fusarium sacchari, Pathogen of Sugarcane Pokkah Boeng Disease
Source: J Fungi (Basel). 2024 Mar 28;10(4):257. doi: 10.3390/jof10040257 (PMC11051555; doi:10.3390/jof10040257)
Supplement: Supplementary file 1 [file jof-10-00257-s001.zip › Table S6.pdf]

Table S6. Effect of *FsBmh* mutation or overexpression on the spores grown on carnation leaf agar.

Strain	Spore length (µm)				Spore width (µm)			
	Microspore (mini)	Microspore (normal)	Macrospore (2 cells)	Macrospore (3-4 cells)	Microspore (mini)	Microspore (normal)	Macrospore (2 cells)	Macrospore (3-4 cells)
WT	8.32±1.52[a]	14.96±2.29[a]	22.10±1.69[ab]	26.90±2.96[b]	1.68±0.45[a]	1.98±0.43[ab]	2.22±0.41[ab]	2.46±0.51[ab]
ΔFsBmh1	8.33±1.23[a]	14.64±2.96[a]	24.55±3.54[ab]	34.91±3.11[a]	1.70±0.41[a]	1.81±0.25[b]	1.94±0.30[b]	2.10±0.25[b]
ΔFsBmh1/sFsBmh2	8.21±1.93[a]	14.12±2.68[a]	25.06±3.08[a]	32.44±2.87[a]	1.74±0.45[a]	1.94±0.18[ab]	2.27±0.46[ab]	2.36±0.53[ab]
O-FsBmh1	8.58±1.24[a]	14.83±1.80[a]	22.75±1.65[ab]	25.17±1.83[b]	1.85±0.31[a]	1.96±0.14[ab]	2.29±0.45[ab]	2.44±0.48[ab]
C-ΔFsBmh1	8.41±2.90[a]	15.25±2.99[a]	22.83±3.04[ab]	25.17±2.23[b]	1.75±0.45[a]	2.08±0.29[ab]	2.20±0.45[ab]	2.42±0.51[ab]
ΔFsBmh2	6.89±1.71[ab]	13.61±2.67[a]	21.60±2.19[b]	25.06±1.67[b]	1.94±0.24[a]	2.33±0.49[ab]	2.60±0.55[a]	2.77±0.42[a]
ΔFsBmh2/sFsBmh1	6.10±1.19[b]	13.44±2.00[a]	21.87±2.42[b]	24.75±1.28[b]	1.85±0.34[a]	2.11±0.33[ab]	2.31±0.46[ab]	2.50±0.53[ab]
O-FsBmh2	8.73±2.24[a]	15.18±1.78[a]	22.73±1.73[ab]	25.36±2.42[b]	1.75±0.40[a]	2.09±0.30[ab]	2.23±0.41[ab]	2.42±0.43[ab]
C-ΔFsBmh2	8.54±3.07[a]	15.09±1.87[a]	22.54±2.16[ab]	25.18±2.44[b]	1.81±0.34[a]	2.04±0.35[ab]	2.25±0.43[ab]	2.42±0.48[ab]
